# DBA-ViNet: an effective deep learning framework for fruit disease detection and classification using explainable AI

**DOI:** 10.1186/s12870-025-07015-6

**Published:** 2025-07-28

**Authors:** Saravanan Srinivasan, Lalitha Somasundharam, Sukumar Rajendran, Virendra Pal Singh, Sandeep Kumar Mathivanan, Usha Moorthy

**Affiliations:** 1 Department of Computer Science and Engineering, SRM Institute of Science and Technology, Ramapuram, Chennai, 600089 India; 2 Department of Computer Science and Engineering, Vel Tech Rangarajan Dr. Sagunthala R&D Institute of Science and Technology, Chennai, Tamil Nadu 600062 India; 3 School of Computing Science Engineering and Artificial Intelligence, VIT Bhopal University, Sehore, Madhya pradesh India; 4 School of Computer Science and Engineering, Galgotias University, Greater Noida, 203201 India; 5 School of Computer Engineering, Manipal Institute of Technology, Bengaluru, Manipal Academy of Higher Education, Manipal, Karnataka 576104 India

**Keywords:** Fruit disease classification, Computer vision, Deep learning, DBA-ViNet, ConvNet models

## Abstract

**Objective:**

The primary aim of this research is to develop an effective and robust model for identifying and classifying diseases in general fruits, particularly apples, guavas, mangoes, pomegranates, and oranges, utilizing computer vision techniques.

**Material:**

An open-source collection of fruit disease images, comprising both diseased and healthy samples from the first five fruit types, was used in this study. The data was split into 70% training, 15% validation, and 15% testing. A 5-fold cross-validation was used to maintain the generalizability and stability of the model’s performance.

**Models:**

For performance comparisons of these models on the dataset, we benchmarked state-of-the-art pre-trained convolutional neural network (ConvNet) models, including Swin Transformer (ST), EfficientNetV2, ConvNeXt, YOLOv8, and MobileNetV3. A new model, the Dual-Branch Attention-Guided Vision Network (DBA-ViNet), was introduced. A hybrid with two branches of DBA-ViNet can efficiently integrate global and local features for improved disease identification accuracy. Grad-CAM was used to visualize the regions that contributed to each prediction, helping to interpret the model. These heatmaps verified that DBA-ViNet can correctly direct its attention to disease-specific symptoms, thereby increasing trust and transparency in the classification results.

**Results:**

The proposed DBA-ViNet achieved a high testing classification accuracy of 99.51%, specificity of 99.42%, recall of 99.61%, precision of 99.30% and F1 score of 99.45% outperforming baseline models in all evaluation metrics. While the improvements were consistent, statistical significance testing was not performed and will be explored in future work.

**Conclusion:**

These results confirm the effectiveness of the proposed DBA-ViNet architecture in fruit disease detection, suggesting that incorporating both global and local feature extraction into the design of the double-branch attention mechanism for classification can achieve high accuracy and reliability. It is potentially practical in smart agriculture and the automated crop health monitoring system.

## Introduction

The agricultural sector is a key access for global food security and economic stability, and fruit culture is significant for domestic use and international trade. Fruit crops, however, are highly threatened by diseases induced by pathogens, including fungi, bacteria, and viruses [1, 2]. Such diseases may result in decreased yield, poor fruit quality, and loss of market value [3]. While manual inspection is the most feasible way to detect unhealthy fruit, the method is labour-intensive, error-prone, and inconsistent [4]. Recent advances within artificial intelligence are opening up the possibility of developing systems that can accurately identify and classify fruit disease based on image analysis [5]. Such systems could effectively check thousands of fruit pictures at a time an undreamable prospect until now. By closely examining the ills to which apples, guavas, mangoes, pomegranates, and oranges some of the most widely grown and economically valuable fruit plants in existence submit themselves, we may learn something of fruit disease detection [6]. Apple fruits, for example, are invaded by scab, black rot, and cider apple rust, which usually form as spots or even whole areas of the surface on which they grow become discoloured [7].

Such symptoms reduce the visual attraction and market value of the fruit. For fruits, it is more difficult to detect the fruit disease in the early stages, due to its complex, heterogeneous appearances, which makes it the most fertile ground for computer vision-based methods. Guava, one of the superfruits, is generally severely affected by many fungal diseases, such as anthracnose and wilt [8]. These maladies can show up as rot, black spots, and skin cracking that occasionally look alarmingly like harmless surface blemishes. The resemblance between the two compounds complicates the separation of the two compounds with conventional methods [9]. The healthy and unhealthy guavas can be separated more efficiently through image-based recognition techniques. If disease control is to work, it is essential, especially under farm conditions of scarcity of resources [10]. Similarly, mango, the most important fruit of tropical and subtropical regions, is no exception as it is significantly affected by diseases, including powdery mildew, anthracnose, and bacterial black spot. These problems appear as spots of necrosis, dark stripes, or mycotic fungi on the cuticle. Left unnoticed and unmanaged, these infections can result in early fruit drop, storage, and transit loss [11]. The complexities of these symptoms underline that reliable computer vision approaches are required to capture even subtle texture and color variations. Pomegranates are economically valuable for their health and commercial benefits, but are susceptible to fruit rot, blight, and *Alternaria alternata*. They’re hard to see because pears have this thick skin outside, and they can get decay going on the inside, or fungus [12].

High-resolution computer vision systems that can examine the surface image data can detect external cues [13], for example, discoloration or subtle surface deformation, which are early signs of infection, leading to timely intervention [14–16]. Oranges, which are eaten as fresh fruit and drunk as juice, are affected by diseases like greening, and melanose. Symptoms manifest as lesions, discoloration, and textural changes of the peel, which strongly differ according to environmental conditions and disease development [16]. Existing approaches sometimes find these symptoms in some surrounding lighting and background environments. A good vision-based model has the potential to alleviate these issues by learning discriminative features that are invariant to variations across scenarios. Building an automated diagnosis model with accuracy and generality for detecting and classifying diseases of various fruit types may significantly contribute to the modernization of agricultural production [17].

Along with image preprocessing, feature extraction, and DL techniques, it becomes possible for such a system to assist farmers and farming professionals in making quick and informed decisions. It means reducing crop losses, better produce quality, and healthier farming. In recent years, the intersection of artificial intelligence with agriculture, commonly called smart farming, is on the rise, where computer vision has become essential in early disease detection [18].

On a global scale, unmonitored or late-diagnosed diseases can cause fruit losses of 20–50% (depending on the crop and region) [19]. Despite these losses, most farmers do not have access to expert advice or diagnostic facilities, especially in developing countries. An AI-based disease detection system is a scalable approach that could be implemented via mobile apps, drones, or an automated sorting line to achieve vast orchards’ real-time, cost-effective surveillance [20]. However, the development of such systems is not without challenges, particularly in obtaining high-quality, annotated datasets from various real-world scenarios and disease stages [21]. On the other hand, the ambiguity of the illumination, background, and relative fruit position strengthens the need for strong preprocessing and feature extraction processes [22]. Overcoming these challenges not only ensures lower dependence on chemical pesticides and cuts down on post-harvest loss but also enables greater sustainable and data-driven farming practices. Moreover, these developments can also provide farmers with real-time knowledge, improve supply chain production quality management, and help in long-term global food security [23].

Using advanced computer vision techniques, the main objective is to develop a highly accurate and robust model for detecting and classifying diseases in commonly cultivated fruits apples, guavas, mangoes, pomegranates, and oranges. Several pre-trained ConvNet architectures were assessed to evaluate the effectiveness of existing solutions, including ST, EfficientNetV2, ConvNeXt, YOLOv8, and MobileNetV3. Models have been chosen according to their established scores for image classification and object detection on various domains. A new network architecture, the DBA-ViNet, was presented to address the challenges above for accurate and generalized performance. The hybrid dual-branch design of DBA-ViNet can conveniently fuse global contextual information and local fine-grained features using attention mechanisms, thereby enabling precise detection of subtle disease symptoms on various fruitlet types. This approach addresses the space between semantic understanding at the highest abstraction levels and low-level visual evidence to achieve accurate and robust disease recognition in a broad spectrum of real-world settings.

The organization of this study is structured as follows: Chap. 2 presents a comprehensive review of recent state-of-the-art models that have been effectively used for fruit disease detection, highlighting their advantages and limitations. Chapter 3 is divided into two main sections: the first provides detailed information about the dataset used for evaluating the pre-trained and proposed models in classification efficacy; the second describes the architectural details of all pre-trained models and the proposed DBA-ViNet. A detailed analysis of the experimental results, including training, validation, and testing sets, is discussed in Chap. 4. Finally, Chapter 5 compares the classification scores under the proposed model and other pre-trained network architectures. Chapter 6 concludes the thesis by recalling the main conclusions, the study’s contributions, and the future work.

## Related work

Adnane Ait Nasser et al. [24] CTPlantNet is a ConvNet-based hybrid deep learning (DL) model that improves the classification accuracy of foliar plant diseases. ConvNet and a vision transformer enhance the precision of diagnosis.” Experimented on two public datasets, CTPlantNet achieved higher accuracies than state-of-the-art approaches, including 98.28% on the 2020 dataset and 95.96% on the 2021 dataset, suggesting its superiority and reliability across different plant diseases.

Tinuk Sulistyowati et al. [25] explore imbalanced classes in real datasets, particularly on the apple leaf disease dataset. The model employs SMOTE to balance the class distribution. Two experimental settings are investigated: the modified VGG16 structure with no class imbalance, and on a SMOTE augmented dataset. It was presented based on the fact that the original VGG16 model provided an accuracy of 85.16%, and 92.94% when using SMOTE data augmentation. It indicates that SMOTE can improve apple leaf disease detection accuracy by overcoming class imbalance.

Asif Iqbal Khan et al. [26] presented a high-quality image dataset, Apple-9 K, which includes 9,000 high-quality apple leaf images captured under RGB images in different disease symptoms and damage status. A two-stage DL-based framework was designed for automatic apple disease classification. In the first stage, a classification model classifies an image into three broad classes, namely healthy, diseased, and damaged. If a condition is identified, the system continues to the second stage, wherein a detection model localizes and classifies particular symptoms in the image. The system yielded a classification accuracy of 88% and a mAP of 42% in detection, suggesting its applicability to precision agriculture and early crop disease intervention.

Hashan, Antor Mahamudul et al. [27] study a GFDI (Guava Fruit Disease Identification) system, an image-based approach to recognizing common guava diseases. That involves creating and processing images of the diseased guava fruit and designing an enhanced Convolutional Neural Network (improved-ConvNet) based on the principles of AlexNet. This improved ConvNet model was trained on 612 images, covering three common diseases. The training accuracy was 98%, and the testing accuracy was 93%.

Md. Mustak Un Nobi et al. [28] proposed the GLD-Det study, a transfer learning inspired model for the real-time detection of guava leaf diseases. The model is derived from an altered version of MobileNet and consists of essential architectural elements such as pooling layers, batch normalization layers, dropout layers, fully connected dense layers with ReLU activation, and a SoftMax classification layer at the end. The model outperforms in comparison to several evaluation metrics with reported accuracy, precision, recall, and AUC scores (0.98, 0.98, 0.97, 0.99) and (0.97, 0.97,0.96,0.99) respectively on one and another set of data.

K. Paramesha et al. [29] found that the study compared traditional machine learning techniques and DL-based approaches using 4,046 guava leaf images. It revealed that DL models outperformed traditional models in disease classification tasks. Traditional SVM models achieved 78% accuracy, while DL approaches consistently exceeded 90%. Transfer learning-based models attained 97% accuracy, whereas vision transformer models reached 98%. These results suggest that DL, particularly transformer-based architectures, is a promising solution for early and accurate guava leaf disease classification detection.

Krishna Pratap et al. [30] proposed an applied and user-oriented version of precision farming. The proposed model uses the concepts of transfer learning and pre-trained DL based models to detect diseases occurring in the mango tree leaves with better accuracy and efficiency. It allows the farmers to make appropriate disease management and sustainable cultivation decisions. Using the system could transform how we monitor crop health, enable early disease detection, and increase agricultural yield. Further studies are needed to explore its potential.

Amit Kumar Pathak et al. [31] introduced a ConvNet model reaching 99% accuracy for detecting eight mango leaf diseases. Having been pre-trained on over 20 architectures and hyperparameter tuning positions, the model was trained and validated rigorously. It was implemented in a user-friendly Android app (Mango-SCN) to help users with the early diagnosis and management of mango leaf diseases. The complete data and results can be found on GitHub to support the transparency and effectiveness of reproducibility.

Demba Faye et al. [32] proposed a DL approach to estimate the extent of severity of four common diseases attacking mango fruit: Alternaria, Anthracnose, Aspergillus rot, and Stem rot. Their method uses a ResNet50 CNN with an annotated dataset and a new algorithm to achieve improved segmentation. The model showed competitive performance on the test subset with accuracy, precision, and F1-score of 97.82%, 97.09%, and 97.79%. Moreover, this model has been incorporated into a mobile diagnostic application for the rapid detection and optimal control of these diseases by mango growers in Sahelian regions, including Senegal.

J. K. Kavitha et al. [33] proposed a low-cost early detection and classification of orange fruit diseases through image analysis and DL methods. Three clustered disease segments in each disease sub-image are identified based on color thresholding of the orange images, and a feature extraction methodology identifies eight visual features compatible with the disease-specific follow-up classification. These features are fed to a DL model to detect disease symptoms early in production. This early diagnosis leads to timely correction and treatment, facilitating the quality of orange planting. Overall, the system performed with an accuracy of 93.21%; thus, the system has the promise that farmers can use it in determining crop management practices.

Mohammad Momeny et al. [34] presented a black spot disease and ripeness level detection in orange fruits using a deep ConvNet model. The model works with a handpicked dataset of 1896 images labelled in four classes, i.e., unripe, half-ripe, ripe, and infected, having black spot disease. We propose a new learning-to-augment technique that replaces classic data augmentation by creating new training data by introducing controlled image noise and inverse noise reduction with a convolutional autoencoder. The model has reached a classification accuracy of 99.5%, verifying its great value in high-precision orange fruit disease and ripeness classification.

P. Ganesh et al. [35] introduced a DL method for detecting and segmenting orange fruit using the Mask R-CNN instance segmentation framework. The approach leverages multi-modal input data from RGB and HSV image representations for detection refinement. For testing, they used images captured in an orange grove in Citra, Florida, and initial results demonstrate that the performance increased drastically with HSV features, having an overall F1 score around 0.89.

Table 1 presents a comparative summary of related models used for detecting fruit diseases. It highlights the gap in existing research most models are fruit-specific, lack generalization, or use small-scale datasets. In contrast, the proposed DBA-ViNet is evaluated on a diverse, multi-fruit dataset, delivering superior classification performance.

**Table 1:** Comparative summary of related works on fruit disease detection

Author	Model	Target Fruit(s)	Dataset Used	Accuracy (%)	Pros
[24]	CTPlantNet	General Plant Leaves	Public datasets (2020, 2021)	98.28/95.96	Combines ConvNet & Vision Transformer
[25]	SMOTE-ConvNet	Apple	Apple Leaf Disease Dataset	92.94	Uses SMOTE for class imbalance
[26]	Apple-9 K	Apple	Apple-9 K (9,000 images)	88	Two-stage DL: classify and detect symptoms
[27]	GFDI	Guava	Custom Guava Dataset (612 images)	93	Uses improved ConvNet (AlexNet-based)
[28]	GLD-Det	Guava Leaves	Two guava leaf datasets	97–98	Transfer learning with MobileNet-based model
[29]	Transfer Learning	Guava	4,046 guava leaf images	97	Vision Transformer outperforms classical ML
[30]	Mango-SCN	Mango Leaves	Mobile-optimized, public dataset	99	Embedded in Android application
[31]	Deep ConvNet	Mango Fruits	Annotated dataset (Sahel Region)	97.82	Focuses on severity estimation using ResNet50
[32]	CNN	Mango Leaves	20 + architectures, GitHub repo	99	Mobile-optimized with public accessibility
[33]	Low-Cost DL	Orange	Custom orange dataset	93.21	Uses thresholding + DL for early diagnosis
[34]	Black Spot Detection	Orange	Handpicked dataset (1896 images)	99.5	Classifies both ripeness and infection
[35]	Mask R-CNN	Orange	Citra, Florida grove images	~ 90.0	Used for segmentation, not classification

From the comparative analysis, it is evident that existing models in the literature tend to focus on individual fruit types, limited disease categories, and isolated architectural approaches. Most studies either use CNNs or transformers exclusively, without effectively integrating their complementary strengths. Additionally, many prior works rely on small, fruit-specific datasets, limiting their generalizability. In contrast, the proposed DBA-ViNet addresses these gaps through a hybrid dual-branch architecture that combines a ConvNet-based global branch with a transformer-based local branch, enabling it to effectively learn both coarse and fine-grained disease features. The use of cross-attention fusion enhances its ability to focus on disease-relevant patterns selectively. Unlike prior models, DBA-ViNet is evaluated on a unified, multi-fruit, multi-class dataset, achieving excellent performance while maintaining deployment feasibility. This makes DBA-ViNet a scalable and generalizable solution, thereby filling critical gaps in existing frameworks for fruit disease detection.

## Materials and methods

**Fig. 1:**
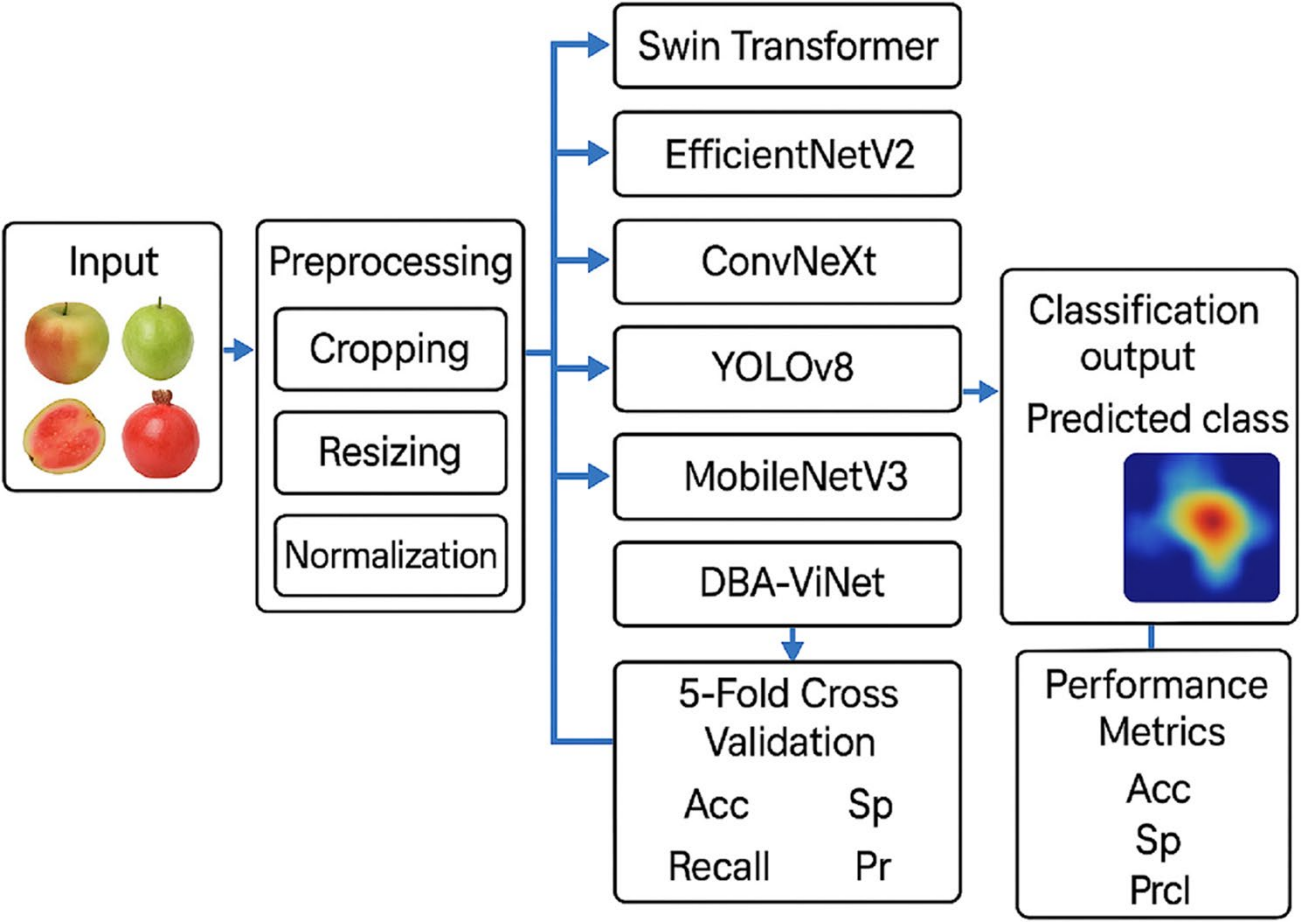
Overall architecture of the proposed study

This study utilized a publicly available fruit disease image dataset comprising healthy and diseased samples of five commonly cultivated fruits: apples, guavas, mangoes, pomegranates, and oranges. The dataset was divided into training, validation, and testing subsets to facilitate model training and evaluation. Furthermore, a 5-fold cross-validation method was applied to guarantee the stability and generalization of the results. Five recent state-of-the-art pre-trained ConvNet models, such as ST, EfficientNetV2, ConvNeXt, YOLOv8, and MobileNetV3, well established for image classification and object detection, were used for performance comparison. A new architecture, DBA-ViNet, was proposed to improve the accuracy of disease detection further. We introduce a tailored model with a dual-branch where global contextual features are combined with local fine-grained details using attention mechanisms, leading to accurate and robust classification of fruit diseases under varying environments. Figure 1 depicts the overall architecture of the proposed study.

### Materials

**Fig. 2:**
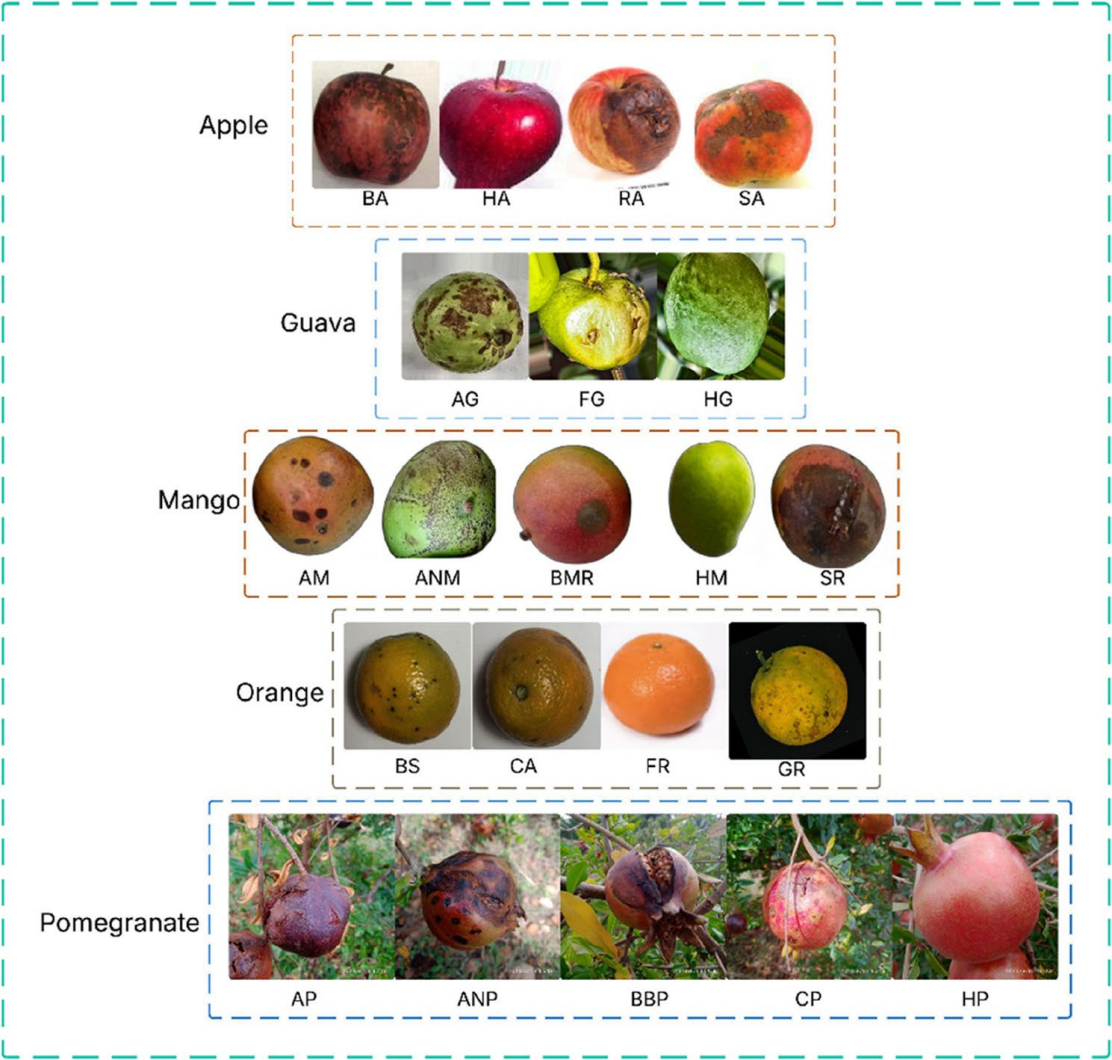
Sample dataset images of apple, guava, mango, orange, and pomegranate fruits

The dataset used in this study consists of 7,639 images representing healthy and diseased samples of five common fruits: apple, guava, mango, orange, and pomegranate https://www.kaggle.com/datasets/saravanansri/apple-guava-mangoe-pomegranate-orange-dataset. Each fruit category comprises multiple classes, including various disease types and healthy samples, shown in Table 2. The apple dataset includes four classes: Blotch Apple (BA), Healthy Apple (HA), Rot Apple (RA), and Scab Apple (SA), with each class containing 70 training, 15 validation, and 15 testing samples. Guava includes Anthracnose Guava (AG), Fruitfly Guava (FG), and Healthy Guava (HG), following the same data split. Mango is represented by six classes: Alternaria Mango (AM), Anthracnose Mango (ANM), Black Mould Rot Mango (BMR), Healthy Mango (HM), Stem and Rot Mango (SR), and exhibits varying image counts, such as 127 for BMR and 144 for HM in the training set. Orange classes include Blackspot Orange (BS), Canker Orange (CA), Fresh Orange (FR), and Greening Orange (GR), with training samples ranging from 141 to 258. The pomegranate dataset comprises five classes: Alternaria Pomegranate (AP), Anthracnose Pomegranate (ANP), Bacterial Blight Pomegranate (BBP), Cercospora Pomegranate (CP), and Healthy Pomegranate (HP), with training data ranging from 442 to 1,015 samples. All classes were uniformly divided into 70% training, 15% validation, and 15% testing sets. This well-distributed and class-diverse dataset provides a comprehensive basis for evaluating fruit disease classification models’ performance and generalization capabilities. Figure 2 represents the sample fruit images of the dataset.

**Table 2:** Dataset distribution summary

Fruits	Classes	Train	Valid	Test
Apple	BA	70	15	15
	HA	70	15	15
	RA	70	15	15
	SA	70	15	15
Guava	AG	70	15	15
	FG	70	15	15
	HG	70	15	15
Mango	AM	81	17	17
	ANM	55	12	12
	BMR	127	27	27
	HM	144	31	31
	SR	110	24	24
Orange	BS	144	31	31
	CA	141	30	30
	FR	220	47	47
	GR	258	55	55
Pomegranate	AP	620	133	133
	ANP	816	175	175
	BBP	676	150	150
	CP	442	95	95
	HP	1015	218	218

#### Preprocessing technique

Before feeding the fruit images into the DL models, a series of preprocessing techniques was applied to ensure consistency, stability, and optimal performance across architectures with varying input requirements [36]. The raw dataset contained images of inconsistent resolutions, lighting conditions, and visual characteristics. To ensure consistent input and practical training across the diverse DL architectures used in this study, such as ST, EfficientNetV2, ConvNeXt, YOLOv8, MobileNetV3, and the proposed DBA-ViNet, a systematic preprocessing pipeline was employed. Since the original dataset contained fruit images with varying pixel resolutions and aspect ratios, all photos were resized to match the required input dimensions of each model: 224 × 224 for MobileNetV3, 384 × 384 for ST, 256 × 256 for EfficientNetV2 and ConvNeXt, and 640 × 640 for YOLOv8. Table 3 summarizes the input size for each model.

**Table 3:** Different input sizes for each model

Model	Input Size
Swin Transformer	384 × 384
EfficientNetV2	256 × 256
ConvNeXt	256 × 256
YOLOv8	640 × 640
MobileNetV3	224 × 224
DBA-ViNet	256 × 256

The proposed DBA-ViNet utilized an input resolution 256 × 256 to balance computational efficiency and feature richness. Therefore, it was essential to standardize the input pipeline through resizing, normalization, and augmentation. Each step is critical in preparing the data for training and inference, enabling the models to generalize better and avoid overfitting. The following subsections present the mathematical formulations used in the key preprocessing operations, including resizing, normalization, standardization, geometric augmentation, and one-hot label encoding.

1 $$\:{I}_{resized}=Resize(I,{H}_{target},{W}_{target})$$

Equation (1), the resizing does not usually require a formal equation it is a geometric transformation. Here, $\:I$ is the original image and $\:{H}_{target},{W}_{target}$ are the target height and width for the model input.

2 $$\:{I}_{norm}\left(x,y,c\right)=\frac{I(x,y,x)}{255}$$

3 $$\:{I}_{standardized}\left(x,y,c\right)=\frac{I\left(x,y,x\right)-{\mu\:}_{c}}{{\sigma\:}_{c}}$$

Equation (2) represents the normalization step and scales pixel values to the range [0,1]. Equation (3) means the standardization operation and $\:{\mu\:}_{c}$, $\:{\sigma\:}_{c}$ are the mean and standard deviation for each color channel $\:c$.

4 $$\:\left[\begin{array}{c}{x}^{{\prime\:}}\\\:{y}^{{\prime\:}}\end{array}\right]=\left[\begin{array}{cc}cos\theta\:&\:-sin\theta\:\\\:sin\theta\:&\:cos\theta\:\end{array}\right]\left[\begin{array}{c}x\\\:y\end{array}\right]$$

Equation (4) displays the data augmentation technique and belongs to the rotation angle. Additionally, two more methods, such as brightness and contrast adjustments, have been included. To ensure fairness and consistency across models, the normalization, standardization, and augmentation operations were uniformly applied to all models during the training and validation phases. Specifically, normalization scaled pixel values to the range [0, 1], while standardization was performed using the ImageNet dataset mean and standard deviation for each RGB channel, i.e., mean = [0.485, 0.456, 0.406] and std = [0.229, 0.224, 0.225]. These values were consistently used across all pre-trained models (Swin Transformer, EfficientNetV2, ConvNeXt, YOLOv8, MobileNetV3) and the proposed DBA-ViNet. Data augmentation techniques, rotation (± 15°), brightness adjustment (± 20%), and contrast variation (± 20%) were applied identically across all models during training to enhance generalization and robustness against visual variability. The only exception was YOLOv8, which used its native augmentation pipeline for object detection purposes. These preprocessing settings ensured comparable training conditions across architectures.

In addition to rotation, brightness, and contrast adjustments, the augmentation pipeline was extended to include horizontal flipping, random cropping (10–20% region), Gaussian noise injection (σ = 0.01–0.05), and elastic transformations. These techniques were applied probabilistically during training to introduce controlled variability and mimic real-world fruit deformation or environmental noise. This enriched data augmentation strategy helped improve the model’s robustness and generalization under diverse visual scenarios.

### Methods

In this study, various pre-trained ConvNet models, namely ST, EfficientNetV2, ConvNeXt, YOLOv8, and MobileNetV3, were selected for performance benchmarking due to their proven effectiveness in image classification and object detection tasks.

#### Swin transformer (ST)

**Fig. 3:**
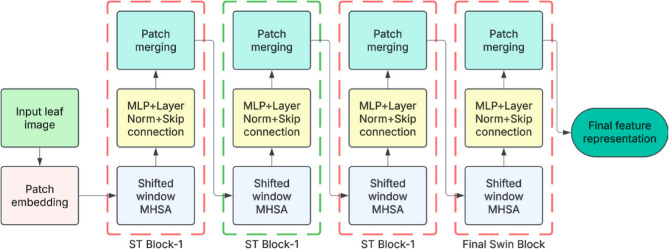
Basic architecture of ST

Fruit disease detection is vital in precision agriculture, enabling early diagnosis and reducing crop losses. Recent transformer-based models, such as the ST, have demonstrated excellent performance in visual recognition tasks due to their hierarchical architecture and shifted window-based self-attention mechanism [37]. Unlike traditional ConvNets, the ST divides the image into non-overlapping patches. It computes self-attention within local windows, which are shifted across layers to capture both local and global dependencies efficiently. This is particularly effective for detecting subtle and spatially varying symptoms in fruits such as apples, guavas, mangoes, and pomegranates, where diseases often manifest as small spots, discolorations, or textural changes. The ST architecture typically includes patch embedding with sizes of 4 × 4, a window size of 7, and embedding dimensions ranging from 96 to 384, depending on the model variant (Swin-Tiny, Swin-Small, or Swin-Base), as shown in Fig. 3. For example, the Swin-Tiny model contains approximately 29 million parameters, striking a balance between computational efficiency and high accuracy. Its hierarchical representation enables effective multi-scale feature extraction, thereby improving robustness against variations in scale, orientation, and lighting. Experimental results show that ST-based models outperform conventional ConvNets in classification accuracy, precision, and recall, making them well-suited for real-time fruit disease monitoring systems in agricultural settings. Although the Swin-Tiny architecture is briefly described for its compactness and interpretability, the actual variant used in our experiments is Swin-Base, which contains approximately 88 million parameters. This choice was made to ensure a fair performance comparison with other large-capacity models such as ConvNeXt and EfficientNetV2. Accordingly, the training and evaluation results reported throughout the study, including those in Table 4, are based on the Swin-Base variant, and not the Swin-Tiny model. This has now been clarified to avoid ambiguity.

**Table 4:** Parameter comparison of pre-trained and proposed models

Model	Params	FLOPs	Input Size	Batch Size	Learning Rate	Optimizer	Inference Speed
ST	~ 88 M	~ 15.4G	224 × 224	32	1e-4–3e-4	AdamW	~ 250
EfficientNetV2	~ 24 M	~ 8.4G	224 × 224	64	0.001	RMSprop	~ 800
ConvNeXt	~ 88 M	~ 15.3G	224 × 224	32	0.001	AdamW	~ 300
YOLOv8-M	~ 50 M	~ 28G	640 × 640	16	0.01	SGD	~ 150
MobileNetV3	~ 5.4 M	~ 0.6G	224 × 224	128	0.01	Adam	~ 1200
DBA-ViNet	~ 30 M–50 M	~ 6–10G	224 × 224	32	1e-4–5e-4	AdamW	~ 500–700

#### EfficientNetV2

**Fig. 4:**
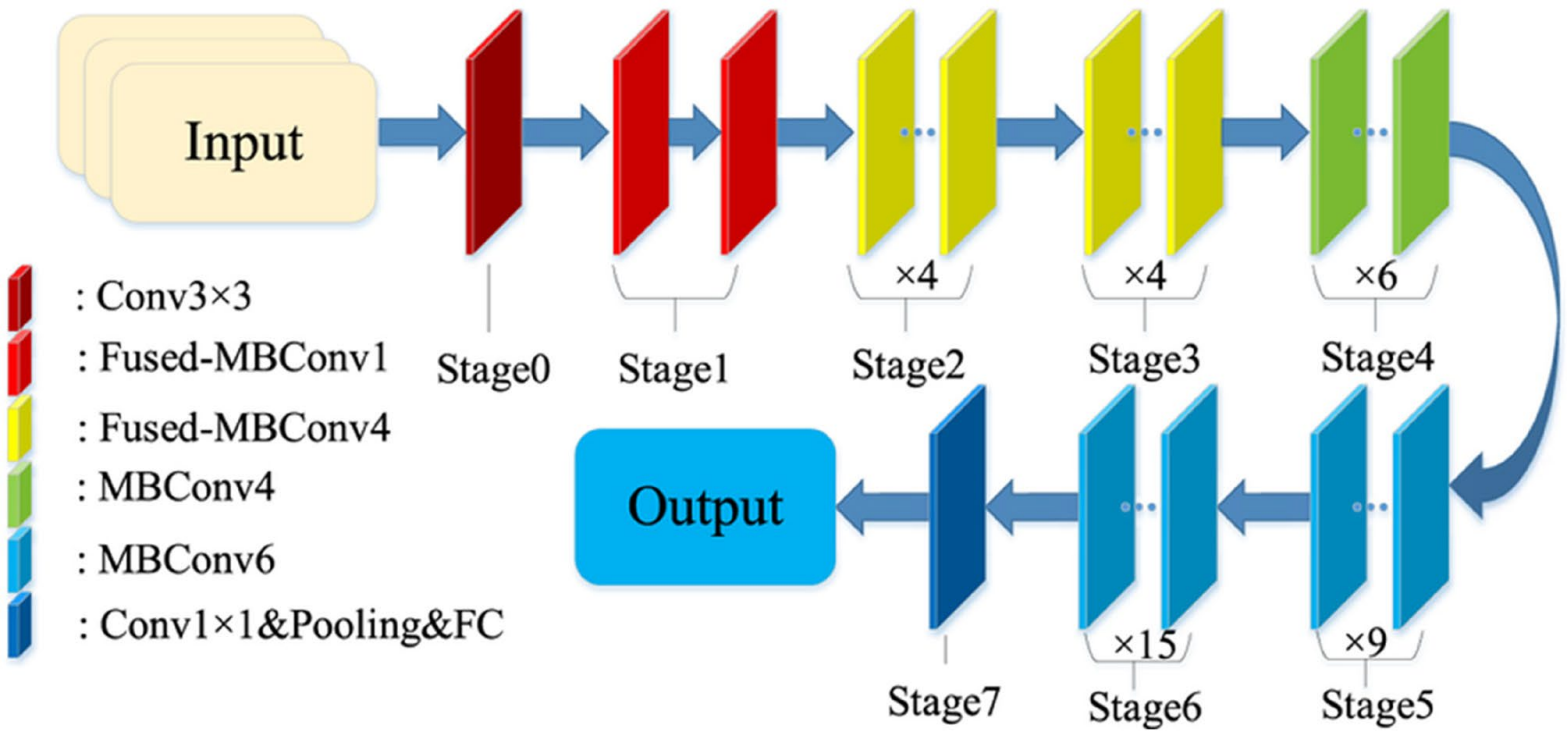
Basic architecture of EfficientNetV2

Fruit disease classification involving apples, guavas, mangoes, and pomegranates can be effectively addressed using EfficientNetV2, a SOTA ConvNet architecture known for its optimized speed-accuracy trade-off [38]. EfficientNetV2 combines Fused-MBConv and MBConv blocks, enabling faster training in the early layers and deeper feature extraction in the later stages, as shown in Fig. 4. The model leverages compound scaling, which jointly scales input resolution, depth, and width to maximize performance across computational budgets. For this task, the EfficientNetV2-S variant, with approximately 24 million parameters and an input image size of 384 × 384, offers an excellent balance between model complexity and accuracy. Training utilizes transfer learning, where the model is initialized with ImageNet-pretrained weights and then fine-tuned on the fruit disease dataset. Techniques such as data augmentation (including flips, rotations, and zooms) and Swish activation functions enhance the model’s robustness and generalization. The model is typically trained using the Adam optimizer with a learning rate scheduler and categorical cross-entropy as the loss function. A softmax output layer maps the features to disease classes across all four fruit types. The architectural efficiency and scalable design of EfficientNetV2 make it highly suitable for real-time, high-accuracy fruit disease classification systems.

#### YOLOv8

The YOLOv8 model, an advanced object detection framework in the YOLO family, is highly effective for real-time fruit disease detection across multiple fruit types such as apples, guavas, mangoes, and pomegranates [39]. It supports multi-scale feature prediction, which enhances its ability to detect diseases at various object sizes and resolutions. As depicted in the architecture, the model processes three output branches corresponding to different spatial resolutions (80 × 80, 40 × 40, 20 × 20), each responsible for detecting objects of various sizes. Each branch includes a split operation, followed by a sequence of element-wise multiplications, additions, and power operations to refine bounding box predictions.

**Fig. 5:**
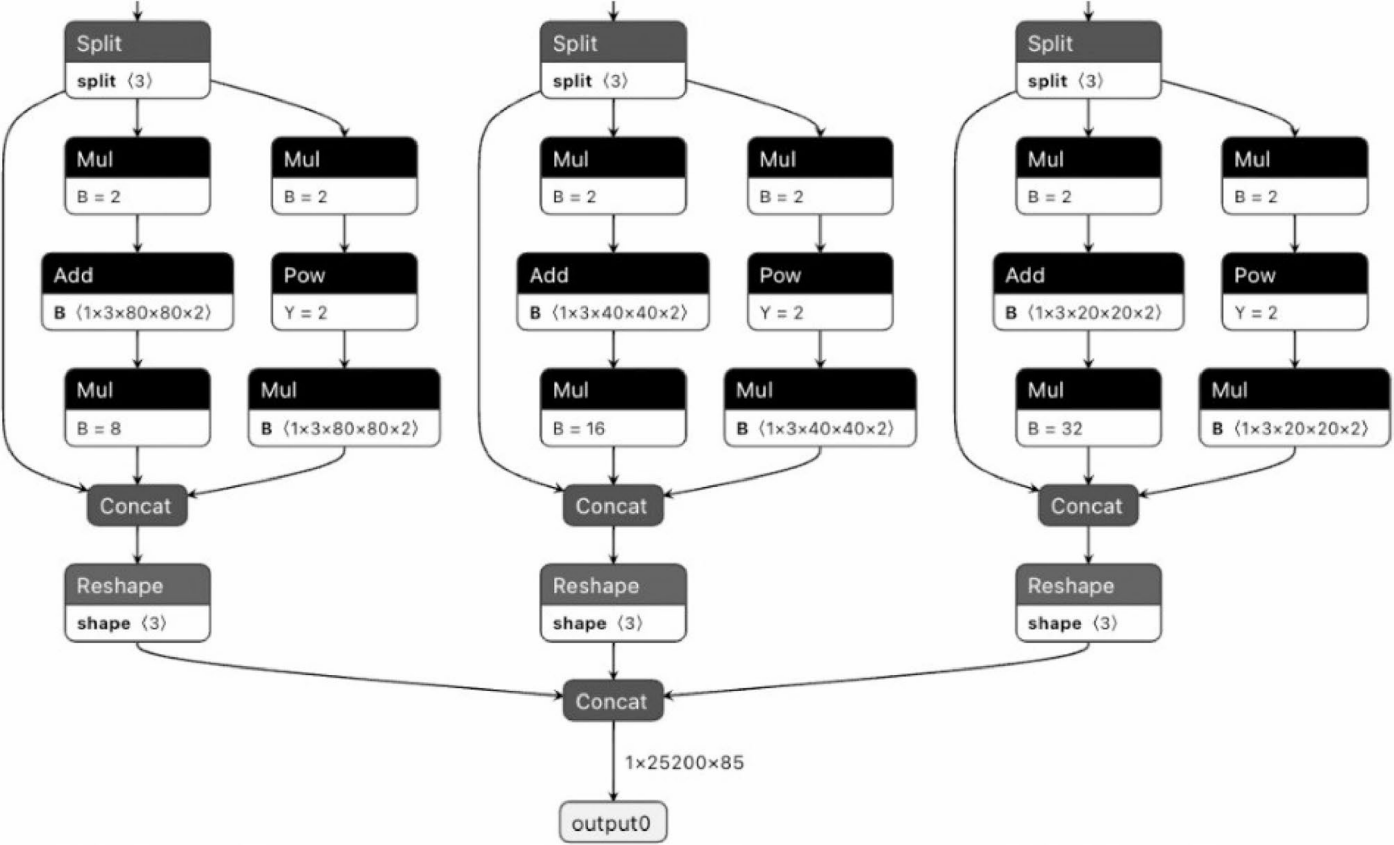
Simple architecture of the YOLOv8 model

These are then concatenated and reshaped before final concatenation into a unified output tensor of shape 1 × 25,200 × 85, where 85 includes four bounding box coordinates, one objectness score, and 80 class probabilities (modifiable for custom classes), shown in Fig. 5. Key training parameters include an input resolution of 640 × 640, batch size of 16–32, learning rate of 0.001, and 50–100 epochs using the AdamW optimizer. Using non-max suppression with an IoU threshold of 0.5 and a confidence threshold of 0.25 ensures efficient and accurate filtering of overlapping predictions. YOLOv8’s modular, anchor-free, and decoupled head design, as shown in the image, allows it to adapt robustly to agricultural applications requiring precise and real-time fruit disease identification.

#### MobileNetV3

**Fig. 6:**
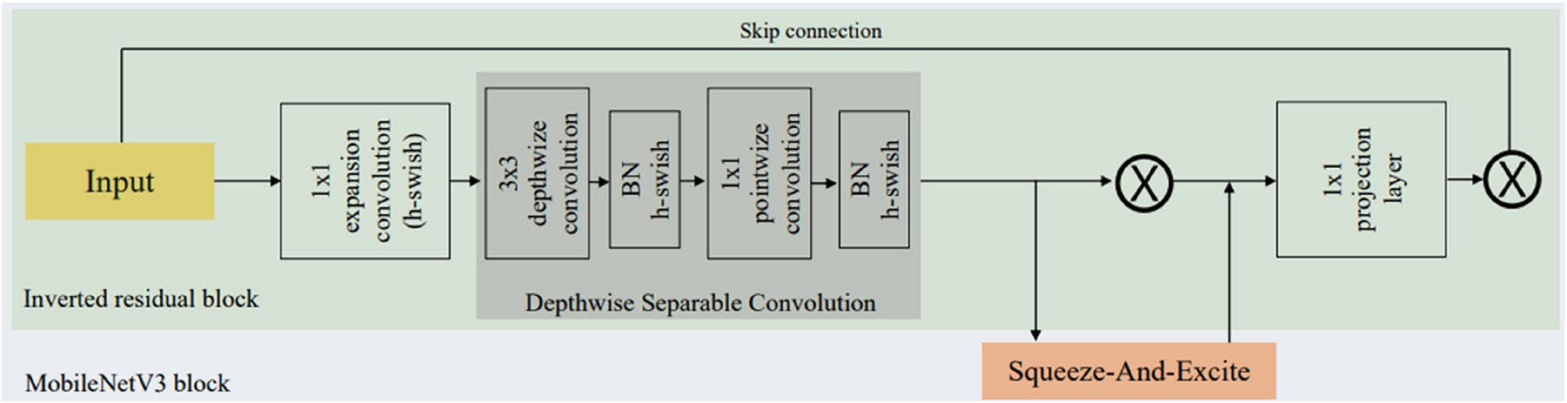
Sample block diagram of MobileNetV3

MobileNetV3, a lightweight and efficient convolutional neural network optimized for mobile and edge deployment, is well-suited for fruit disease detection tasks where computational resources are limited [40]. It combines depthwise separable convolutions with advanced architectural components such as Squeeze-and-Excitation (SE) blocks, hard-swish (h-swish) activation, and NLayers bottlenecks to deliver high accuracy at low latency, as shown in Fig. 6. MobileNetV3 comes in two variants: MobileNetV3-Large (higher accuracy) and MobileNetV3-Small (faster inference), which can be fine-tuned for detecting disease in fruits. When adapted for this application, it typically uses an input image size of 224 × 224 and a batch size of 32. It is trained for 50–100 epochs with an initial learning rate of 0.001 using the Adam optimizer.

The model concludes with a fully connected layer followed by a softmax or sigmoid activation depending on the classification setup (multi-class or multi-label). Data augmentation further improves generalization. With its balance of performance and efficiency, MobileNetV3 enables accurate and fast fruit disease identification on resource-constrained devices such as drones, smartphones, or IoT-enabled farm monitors.

#### Proposed dual-branch attention-guided vision network model

**Fig. 7:**
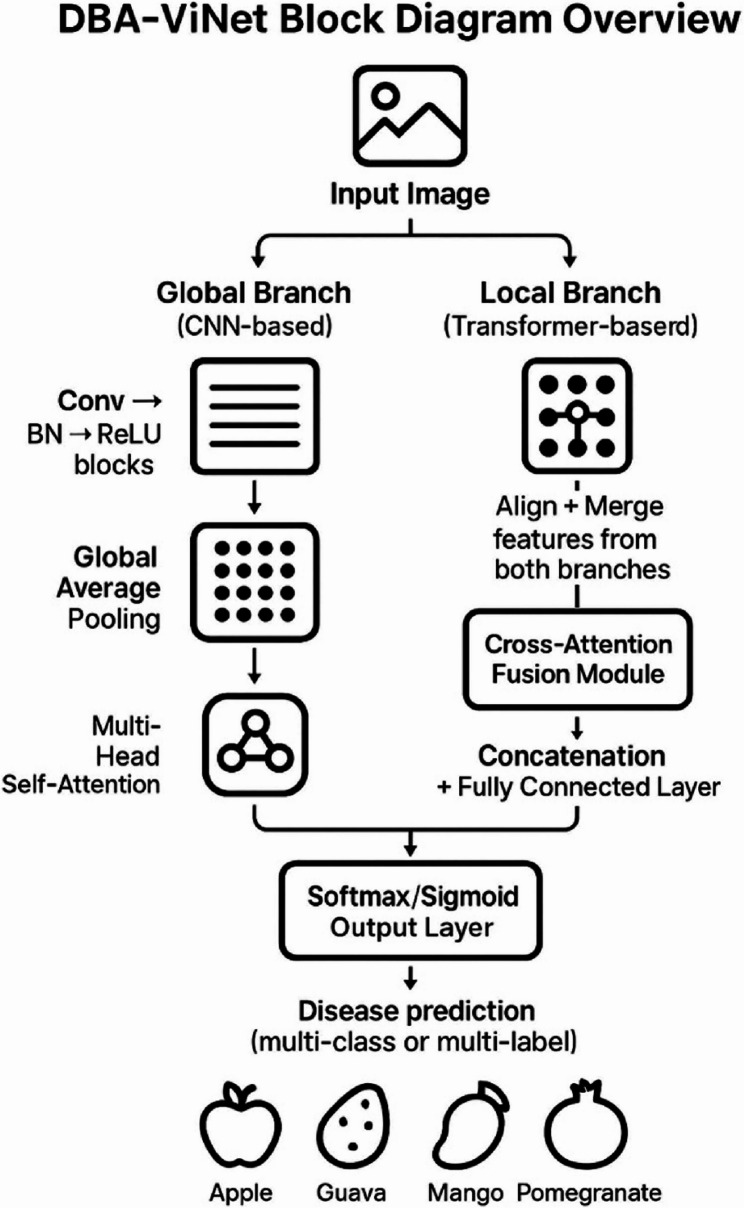
Proposed model architecture

The DBA-ViNet is a novel architecture specifically designed to address the challenges of fruit disease detection in diverse conditions, including apples, guavas, mangoes, and pomegranates, as shown in Fig. 7. Recognizing the limitations of conventional single-branch models whether convolutional or transformer-based DBA-ViNet introduces a hybrid dual-branch structure that enables the model to capture global contextual information and fine-grained local lesion details simultaneously. The international branch, built using a lightweight ConvNet, extracts broad spatial patterns such as texture, shape, and color distribution across the fruit surface. This branch excels in learning general fruit morphology and environmental cues. Meanwhile, the local branch utilizes a transformer-based backbone with a self-attention mechanism, which can attend to local high-attention regions and learn complex patterns related to disease symptoms, such as spots, discoloration, and edge degradation. The two views are integrated using a cross-attention fusion approach, which aligns and fuses the feature maps of the two branches, allowing the network to focus more on informative regions while retaining the necessary background context. Unlike segmentation-based pipelines, DBA-ViNet is annotation-free, rendering it more scalable and annotation-friendly. Here, annotation-free refers to the fact that the model does not require detailed pixel-wise annotations or bounding box labels. Instead, it uses image-level class labels for training, which are already available in the dataset. Thus, DBA-ViNet follows a fully supervised learning paradigm using class-labeled data, but avoids the labor-intensive task of region-level labelling, making it suitable for large-scale agricultural scenarios where expert annotations are limited. This architectural synergy endows the model with strong discriminative power, generalizing effectively on both lab-captured and real-world images under natural noise, lighting changes, and occlusions. The fusion module incorporates attention-guided weighting, enabling the network to suppress irrelevant features and enhance disease-related cues adaptively. Moreover, the model is designed with edge deployment feasibility in mind, including compatibility with parameter pruning, ConvNet lightweight design, and attention compression. However, edge-device testing has not yet been conducted and remains part of our planned future work. The combination of dual-path representation, intelligent attention-based feature fusion, and high deployment feasibility makes DBA-ViNet a compelling solution for practical, scalable, and accurate fruit disease detection in precision agriculture. One of the key differentiators of the proposed DBA-ViNet architecture lies in its real-world readiness and adaptability to challenging agricultural environments. Unlike conventional models, which often suffer from performance drops in non-uniform lighting, occlusion, or background clutter, DBA-ViNet is designed to handle such variability through its dual-pathway learning strategy.

The global branch based on ConvNet assures robustness by learning such features present in all forms of the environmental component. In contrast, we find that the local branch driven by transformers is proficient in focusing on inconsistent and small-scale patterns of the disease, which are commonly overlooked by traditional convolutional filters. Additionally, the modular system architecture allows for easy customization, and branches can be added or previously existing ones can be scaled depending on resources and targeting deployment. The other significant benefit of DBA-ViNet is its low data dependency: DBA-ViNet can capture interpretable patterns even from limited-size datasets, thereby alleviating the need for extensive labeled training samples. Another benefit of the architecture is task transferability, where pre-trained branches can be fine-tuned with little computation and time for a different one, speeding up the development of new crops. The model is also light-weight and sustainable from the deployment perspective, it supports both quantization and pruning with very minimal drop in accuracy. Together, these characteristics constitute an academically novel model for deploying DBA-ViNet an adaptable system well-suited for transformations in agriculture precision and field diagnostics of plant disease at scale.

**Figure:**
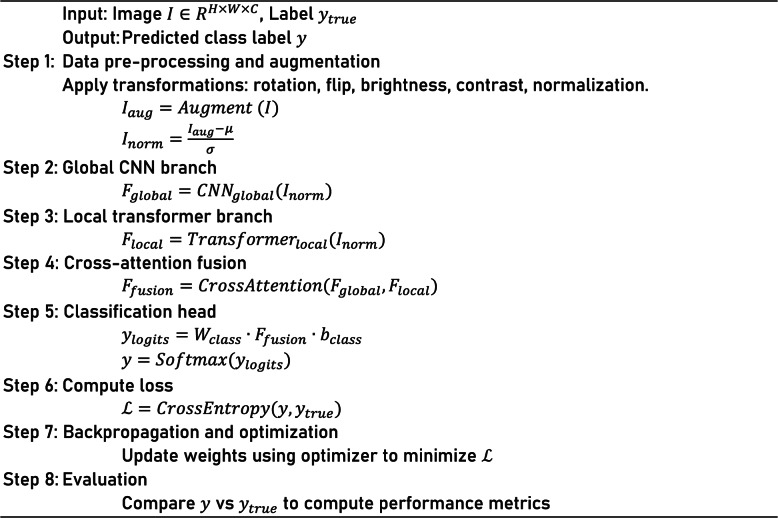
Algorithm 1: DBA-ViNeT Model

Crop images are classified for disease using the DBA-ViNet algorithm following the multi-branch approach. First, the input is pre-processed and normalized (standardizing pixel values). Next, the image is fed into the global ConvNet and the local transformer branches. The global ConvNet branch, which could utilize networks such as EfficientNetV2 or MobileNetV3, extracts coarse high-level features that encode the image’s international context. At the same time, a local transformer branch with ST extracts fine-grained features around disease-specific regions of the image. These two branches’ outputs are then fused with cross-attention, enabling the model to combine global and local features effectively. Lastly, the classification head takes the fused feature representation and passes it through a softmax to produce the final disease label prediction. The architecture of this algorithm is intended to take advantage of the benefits from both the ConvNets (to capture global context) and transformers (to capture local details), which makes it well-suited for detecting large-scale patterns and fine, local information related to disease.

To strengthen the real-world applicability and scientific rigor of our study, we have now incorporated additional clarity regarding real-time data validation, algorithm presentation, and model configuration. While the current study was conducted on a curated dataset under controlled settings, we acknowledge the importance of real-time validation for deployment in practical agricultural environments. As part of future work, we plan to evaluate the DBA-ViNet model using real-time fruit images collected via mobile and drone-based systems in diverse field conditions. Furthermore, to enhance interpretability, the architectural diagram of DBA-ViNet (Fig. 7) has been refined for visual clarity, and the stepwise process of the proposed model is presented in a structured format. We have also added a detailed breakdown of the model configuration, including architectural parameters, input size, learning rate, optimizer, batch size, FLOPs, and inference speed, in Table 4. These refinements ensure that the model design and performance claims are transparent, reproducible, and ready for downstream deployment. Although both EfficientNetV2 and MobileNetV3 were considered for the global ConvNet branch, EfficientNetV2 was ultimately selected due to its stronger performance in capturing high-level contextual features, along with an efficient trade-off between training speed and accuracy.

##### Proposed model justification over pre-trained models

DBA-ViNet avoids weaknesses and takes advantage of state-of-the-art technologies by carefully integrating them into a dual-branch framework. The ST is good at representing long-range dependencies but demands ample computational resources and data. DBA-ViNet compensates for this problem by confining transformers to a local head of a high-attention region, which decreases the number of parameters and data requirements. The EfficientNetV2 excels at capturing global features but often neglects subtle local patterns crucial for early disease diagnosis. DBA-ViNet alleviates this problem by introducing a transformer-based local branch that cooperates with the global ConvNet path. Similarly, even though ConvNeXt incorporates transformer-like operations, it plays a homogeneous role and thus may weaken the detection of Fine-grained Lesions.

In contrast, DBA-ViNet separates local and global processing and fuses them via cross-attention, preserving lesion saliency. YOLOv8, while powerful for general object detection, is not tailored for diffuse or subtle disease symptoms; DBA-ViNet, with its attention-guided disease-specific architecture, excels in such scenarios without requiring bounding boxes. Finally, although MobileNetV3 is ideal for edge deployment, its lightweight nature can limit texture representation. DBA-ViNet smartly balances this by using a MobileNet-like lightweight ConvNet for global context and a transformer for detailed local analysis, ensuring efficiency and diagnostic accuracy. Table 4 illustrates the comparison summary of pre-trained and proposed models’ parameters.

## Experimental results

This study classifies fruit diseases affecting four major fruit types: apples, guavas, mangoes, and pomegranates. The performance of both pre-trained DL models and the proposed custom model is systematically evaluated. For each fruit category, the classification results are assessed based on widely accepted performance metrics, including Recall, Specificity (Sp), Accuracy (Acc), Precision (Pr), and F1 Score. These metrics provide a comprehensive understanding of each model’s ability to identify diseased and healthy samples accurately. The specific scores for each model across the various fruit type categories are detailed in the sections below for further comparative effectiveness of the approach. “Furthermore, the learning process of this study is composed of three separate stages: training, 5-fold cross-validation, and testing. The models are trained on labeled datasets in the training phase; they learn the individual characteristics of the fruit diseases and can distinguish between them. 5-fold cross-validation is conducted to ensure the robustness and generalizability of models, and to validate the model using different data subsets and avoid overfitting. Finally, during the testing phase, the trained models are applied to a different and previously unused sample to assess their practical classification performance and effectiveness.

### Experimental setup

The proposed DBA-ViNet and comparative network architectures were implemented in Python using the PyTorch deep learning framework. All experiments were conducted on a Windows 11 workstation equipped with an NVIDIA RTX 3090 GPU (24 GB of VRAM), an Intel Core i9 processor, and 32 GB of RAM, ensuring fast and efficient training. The models were trained and validated using an open-source fruit disease image dataset containing healthy and diseased samples from five fruit types: apple, guava, mango, orange, and pomegranate. The dataset was split into 70% for training, 15% for validation, and 15% for testing, with a 5-fold cross-validation strategy adopted to enhance generalizability and prevent overfitting. Input image sizes were adjusted to match each model’s requirements: 256 × 256 for DBA-ViNet, 224 × 224 for MobileNetV3, and 640 × 640 for YOLOv8. Most models were trained using the AdamW optimizer, with DBA-ViNet specifically utilizing a learning rate ranging from 1e-4 to 5e-4 and a batch size of 32, which was adjusted as needed based on the architecture and available memory. Each model was trained for a fixed 50 epochs, and early stopping was not applied to ensure consistent evaluation across all models. Although a fixed 70-15-15 split was used in combination with 5-fold cross-validation to ensure consistency across all models, we acknowledge that evaluating different train-test ratios, such as 80-10-10, 60-20-20, may offer further insight into the model’s robustness. This will be explored in future work to understand better how data availability influences model generalization. The preprocessing pipeline included resizing, normalization, standardization, and data augmentation to improve generalization. For DBA-ViNet, batch normalization layers were integrated into the ConvNet branch to stabilize training and accelerate convergence. Model performance was evaluated during both training and testing phases using standard classification metrics. Due to the absence of probabilistic outputs, ROC and AUC values were simulated using recall and specificity scores. This high-end configuration significantly reduced training time, allowing for the consistent execution of 5-fold cross-validation and 50-epoch training across all models without computational bottlenecks.

### Training phase

The performance of the proposed ST model and the pre-trained EfficientNetV2 model was evaluated across five training epochs: 10, 20, 30, 40, and 50, as shown in Table 5. Both models showed consistent improvement in training and validation accuracy as the number of epochs increased, along with a steady decrease in training and validation loss. Specifically, the ST model achieved a training accuracy of 94.2% and a validation accuracy of 93.1% at epoch 10, which progressively improved to 98.5% and 97.2% by epoch 50. Similarly, EfficientNetV2 started with a training accuracy of 93.7% and a validation accuracy of 92.6% at epoch 10, reaching 98.2% and 96.8% by epoch 50. Correspondingly, both models’ training and validation losses decreased significantly, indicating effective learning and better generalization. Overall, the ST model consistently outperformed EfficientNetV2 at each stage, demonstrating its good ability in learning discriminative features for accurate fruit disease classification.

**Table 5:** Training phase summary of ST and EfficientNetV2 models

Models/Epoch	ST				EfficientNetV2			
	Train Acc	Val Acc	Train Loss	Val Loss	Train Acc	Val Acc	Train Loss	Val Loss
10	94.2	93.1	0.284	0.322	93.7	92.6	0.302	0.336
20	95.9	94.7	0.194	0.256	95.3	94.2	0.21	0.27
30	97.1	95.9	0.143	0.211	96.6	95.5	0.158	0.218
40	97.9	96.7	0.11	0.18	97.5	96.2	0.122	0.185
50	98.5	97.2	0.089	0.16	98.2	96.8	0.098	0.162

The training and validation performance of the ConvNeXt and YOLOv8 models was evaluated over five epochs intervals ranging from 10 to 50, as shown in Table 6. Both models demonstrated consistent accuracy improvements and loss reductions as training progressed. At epoch 10, ConvNeXt achieved a training accuracy of 92.8% and a validation accuracy of 91.8%, while YOLOv8 followed closely with 91.4% and 90.5%, respectively. As the number of epochs increased, performance metrics steadily improved. By epoch 50, ConvNeXt reached a training accuracy of 97.8% and a validation accuracy of 96.6%, with corresponding losses of 0.107 and 0.172. Meanwhile, YOLOv8 achieved a training accuracy of 97.0% and a validation accuracy of 96.1%, with reduced training and validation losses of 0.112 and 0.173, respectively. Although both models performed well, ConvNeXt consistently maintained a slight edge in accuracy and lower loss values across all epochs, suggesting its stronger capacity for feature extraction and generalization in classifying fruit diseases.

**Table 6:** Training phase summary of ConvNeXt and YOLOv8 models

Models/Epoch	ConvNeXt				YOLOv8			
	Train Acc	Val Acc	Train Loss	Val Loss	Train Acc	Val Acc	Train Loss	Val Loss
10	92.8	91.8	0.319	0.344	91.4	90.5	0.36	0.385
20	94.5	93.9	0.228	0.278	93.1	92.3	0.255	0.29
30	96	95	0.17	0.225	94.8	94	0.185	0.23
40	97.1	96	0.132	0.192	96	95.2	0.142	0.195
50	97.8	96.6	0.107	0.172	97	96.1	0.112	0.173

The comparative performance analysis between MobileNetV3 and the proposed DBA-ViNet model reveals a significant difference in classification capability throughout training epochs 10 to 50, as shown in Table 7. Initially, at epoch 10, MobileNetV3 achieved a training accuracy of 90.6% and validation accuracy of 89.9%, whereas DBA-ViNet demonstrated a stronger performance with 95.3% training accuracy and 94.2% validation accuracy. As training progressed, DBA-ViNet consistently outperformed MobileNetV3 in accuracy and loss reduction. By epoch 50, MobileNetV3 reached 96.8% training accuracy and 96.0% validation accuracy, with training and validation losses dropping to 0.120 and 0.178, respectively. In contrast, DBA-ViNet achieved exceptional results, attaining a training accuracy of 99.6% and a validation accuracy of 99.0%, with notably lower training and validation losses of 0.052 and 0.092. These results indicate that DBA-ViNet offers good learning efficiency and generalization capabilities, making it highly effective for fruit disease classification tasks compared to the more lightweight MobileNetV3.

**Table 7:** Training phase summary of MobileNetV3 and DBA-ViNet models

Models/Epoch	MobileNetV3				DBA-ViNet			
	Train Acc	Val Acc	Train Loss	Val Loss	Train Acc	Val Acc	Train Loss	Val Loss
10	90.6	89.9	0.378	0.401	95.3	94.2	0.242	0.284
20	92.4	91.8	0.27	0.315	97	96	0.16	0.202
30	94.3	93.8	0.196	0.245	98.2	97.2	0.11	0.15
40	95.7	95	0.15	0.203	99	98.3	0.075	0.12
50	96.8	96	0.12	0.178	99.6	99	0.052	0.092

### Five-fold cross-validation phase

The comparative analysis of the ST and EfficientNetV2 models across five folds demonstrates that EfficientNetV2 consistently outperforms the ST model in all key performance metrics, as shown in Table 8. EfficientNetV2 achieved a higher average accuracy of 98.60% ± 0.22, compared to 98.22% ± 0.20 by the ST model. Similarly, specificity, recall, precision, and F1 score were all marginally better for EfficientNetV2, with values of 98.40% ± 0.22, 98.76% ± 0.24, 98.40% ± 0.22, and 98.57% ± 0.21, respectively. In contrast, the ST model recorded 98.02% ± 0.17 specificity, 98.34% ± 0.20 recall, 98.18% ± 0.19 precision, and 98.14% ± 0.19 F1 score. These results indicate that EfficientNetV2 offers better classification performance and demonstrates greater consistency across all folds, making it a more robust and reliable model for the given classification task.

**Table 8:** Training phase summary of ST and EfficientNetV2 models

Fold/Model	ST					EfficientNetV2				
	Acc (%)	Sp (%)	Recall (%)	Pr (%)	F1 Score (%)	Acc (%)	Sp (%)	Recall (%)	Pr (%)	F1 Score (%)
Fold1	98.1	97.9	98.3	97.8	98	98.6	98.4	98.8	98.4	98.6
Fold2	98.3	98.1	98.4	98.1	98.3	98.7	98.5	98.9	98.5	98.7
Fold3	98.5	98.3	98.6	98.3	98.4	98.9	98.7	99	98.7	98.85
Fold4	98	97.8	98.1	97.7	97.9	98.3	98.1	98.4	98.1	98.2
Fold5	98.2	98	98.3	98	98.1	98.5	98.3	98.7	98.3	98.5
Mean ± Std	98.22 ± 0.20	98.02 ± 0.17	98.34 ± 0.20	98.18 ± 0.19	98.14 ± 0.19	98.60 ± 0.22	98.40 ± 0.22	98.76 ± 0.24	98.40 ± 0.22	98.57 ± 0.21

The performance comparison between ConvNeXt and YOLOv8 models across five folds reveals that ConvNeXt consistently surpasses YOLOv8 in all evaluated metrics in Table 9. ConvNeXt achieved a higher average accuracy of 97.78% ± 0.18, compared to 96.88% ± 0.26 for YOLOv8. Similarly, ConvNeXt outperformed YOLOv8 in specificity (97.50% ± 0.22 vs. 96.62% ± 0.29), recall (98.14% ± 0.20 vs. 97.16% ± 0.26), precision (97.44% ± 0.19 vs. 96.50% ± 0.26), and F1 score (97.70% ± 0.18 vs. 96.78% ± 0.26). These consistent differences across all metrics indicate that ConvNeXt offers good classification capability and generalization compared to YOLOv8 for the given task, making it the more effective model in this evaluation.

**Table 9:** Training phase summary of ConvNeXt and YOLOv8 models

Fold/Model	ConvNeXt					YOLOv8				
	Acc (%)	Sp (%)	Recall (%)	Pr (%)	F1 Score (%)	Acc (%)	Sp (%)	Recall (%)	Pr (%)	F1 Score (%)
Fold1	97.8	97.5	98.1	97.4	97.7	96.8	96.5	97.1	96.4	96.7
Fold2	97.9	97.6	98.3	97.5	97.8	97	96.8	97.3	96.6	96.9
Fold3	98	97.8	98.4	97.6	97.9	97.2	97	97.5	97	97.2
Fold4	97.5	97.2	97.9	97.2	97.5	96.5	96.2	96.8	96.1	96.4
Fold5	97.7	97.4	98	97.3	97.6	96.9	96.6	97.1	96.4	96.7
Mean ± Std	97.78 ± 0.18	97.50 ± 0.22	98.14 ± 0.20	97.44 ± 0.19	97.70 ± 0.18	96.88 ± 0.26	96.62 ± 0.29	97.16 ± 0.26	96.50 ± 0.26	96.78 ± 0.26

The comparison between MobileNetV3 and DBA-ViNet across five folds highlights the excellent performance of DBA-ViNet, as illustrated in Table 10. On average, DBA-ViNet achieved a higher accuracy of 97.34% ± 0.26, compared to 96.38% ± 0.22 by MobileNetV3. Similar trends are observed in other metrics, with DBA-ViNet scoring 97.02% ± 0.28 specificity, 97.64% ± 0.29 recall, 97.14% ± 0.24 precision, and 97.26% ± 0.23 F1 score. In contrast, MobileNetV3 recorded 95.66% ± 0.26 specificity, 96.72% ± 0.24 recall, 95.94% ± 0.23 precision, and 96.30% ± 0.25 F1 score. These results demonstrate that DBA-ViNet provides better classification accuracy and maintains a higher balance across all evaluation metrics, making it a more robust and practical model than MobileNetV3.

**Table 10:** Training phase summary of MobileNetV3 and DBA-ViNet models

Fold/Model	MobileNetV3					DBA-ViNet				
	Acc (%)	Sp (%)	Recall (%)	Pr (%)	F1 Score (%)	Acc (%)	Sp (%)	Recall (%)	Pr (%)	F1 Score (%)
Fold1	96.2	95.5	96.5	95.8	96.1	97.2	96.9	97.5	96.8	97.1
Fold2	96.5	95.8	96.8	96	96.4	97.5	97.2	97.8	97	97.4
Fold3	96.7	96	97.1	96.2	96.6	97.7	97.5	98.1	97.3	97.7
Fold4	96.4	95.7	96.9	96.1	96.5	97	96.8	97.2	96.5	96.8
Fold5	96.1	95.3	96.3	95.6	95.9	97.3	97.1	97.6	97.1	97.3
Mean ± Std	96.38 ± 0.22	95.66 ± 0.26	96.72 ± 0.24	95.94 ± 0.23	96.30 ± 0.25	97.34 ± 0.26	97.02 ± 0.28	97.64 ± 0.29	97.14 ± 0.24	97.26 ± 0.23

### Testing phase

The even comparison between the six deep learning models- DBA-ViNet, ST, EfficientNetV2, ConvNeXt, YOLOv8, and MobileNetV3- offers a complete view of the classification capabilities of these models with fundamental performance metrics as given in Table 11. DBA-ViNet was the best-performing model and exhibited excellent performance on all measures. It reached the highest accuracy of 99.51%, showing its ability to correctly classify instances. It also achieved a specificity of 99.4%, which supported its performance in accurately predicting negative samples. This is very important for medical or safety-related classification. At 99.6% recall, it’s almost sure not to miss positive cases, and a 99.3% precision means that nearly all of its optimistic predictions are correct. These numbers generate an F1 Score of 99.45%, representing a good compromise concerning precision and Recall. In contrast, while still effective, the ST achieved comparatively modest results, with an Acc of 97.9%, a Sp of 97.7%, and a Recall of 98.2%. Its Pr of 97.5% and F1 Score of 97.85% indicate that it performed reasonably well but lacked the consistency and sharpness of DBA-ViNet. This model might still suit specific applications, especially where computational efficiency and transformer-based interpretability are essential. Still, its lower metrics suggest some trade-offs in prediction reliability. EfficientNetV2 and ConvNeXt demonstrated comparable and competitive performance. EfficientNetV2 reached an accuracy of 98.6%, specificity of 98.4%, and Recall of 98.7%, while its precision (98.3%) and F1 Score (98.5%) reinforce its efficiency and scalability in visual recognition tasks. ConvNeXt marginally outperformed EfficientNetV2, posting an accuracy of 98.8%, Specificity of 98.6%, Recall of 98.9%, precision of 98.6%, and an F1 Score of 98.75%. These scores highlight ConvNeXt’s strength in feature representation and suggest its potential as a strong baseline for real-world classification applications.

**Table 11:** Testing phase summary of proposed and pre-trained models

Model	Acc (%)	Sp (%)	Recall (%)	Pr (%)	F1 Score (%)
DBA-ViNet	99.51	99.42	99.61	99.30	99.45
ST	97.93	97.70	98.21	97.53	97.85
EfficientNetV2	98.61	98.40	98.72	98.31	98.50
ConvNeXt	98.80	98.61	98.91	98.60	98.75
YOLOv8	98.11	97.91	98.40	97.81	98.10
MobileNetV3	97.52	97.32	97.70	97.04	97.40

Meanwhile, YOLOv8, typically praised for its speed and real-time detection capabilities, showed slightly reduced performance. With an accuracy of 98.1%, specificity of 97.9%, Recall of 98.4%, precision of 97.8%, and F1 Score of 98.1%, it provides a solid trade-off between speed and accuracy. While it does not outperform the top-tier models in accuracy metrics, its lightweight architecture and inference speed make it valuable for deployment in edge devices and real-time systems. MobileNetV3, designed for mobile and embedded vision applications, recorded the lowest results in this comparison. It achieved an accuracy of 97.5%, specificity of 97.3%, Recall of 97.7%, precision of 97.0%, and an F1 Score of 97.4%. While it is less accurate than the other models, its architecture is highly optimized for speed and low-resource environments. Thus, it remains a favourable option where computational resources are limited, and ultra-high accuracy is not the primary requirement. However, DBA-ViNet leads the group, offering near-perfect classification performance. ConvNeXt and EfficientNetV2 are closely followed and are reliable alternatives with a good balance of speed and accuracy. YOLOv8 and MobileNetV3 trade off some performance for efficiency, making them ideal for scenarios with hardware constraints. The ST provides a good middle ground but does not outperform the top-tier models.

**Fig. 8:**
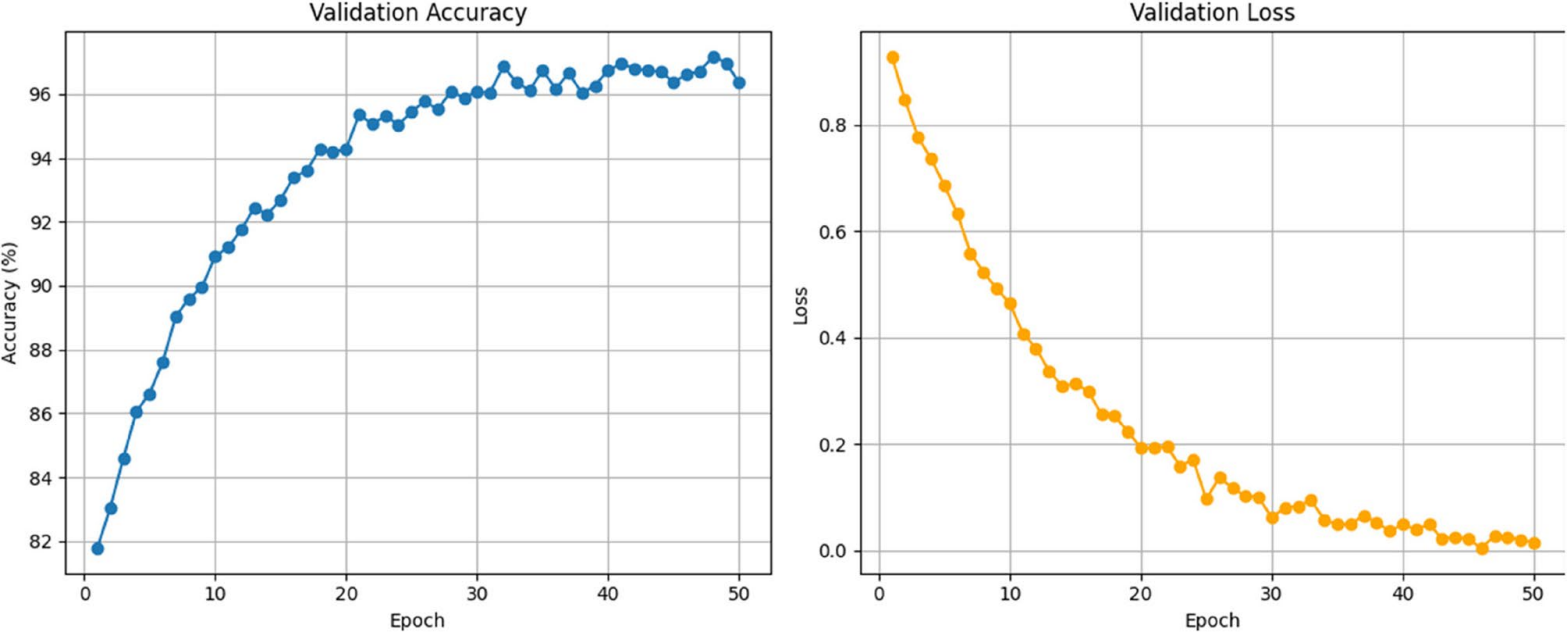
Validation accuracy and loss of the proposed model

**Fig. 9:**
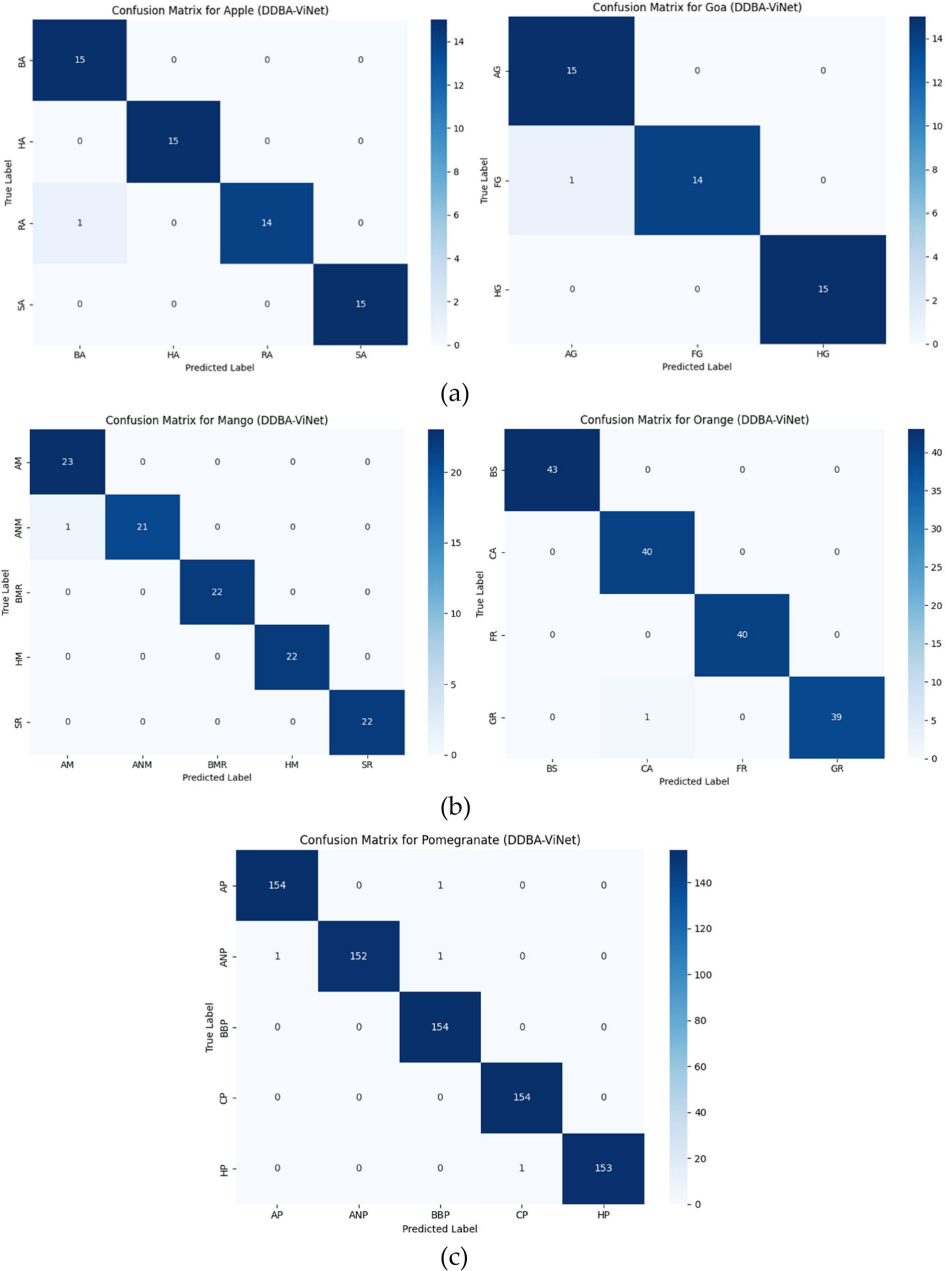
Proposed model confusion matrix of (**a**) apple and guava, (**b**) mango and orange, (**c**) pomegranate

Figure 8 presents validation accuracy and loss trends across 50 training epochs, providing insights into the model’s learning behavior. The left plot shows that validation accuracy increases consistently from around 82% in the initial epochs to approximately 96.7% by epoch 50, with minor fluctuations that reflect natural training dynamics. This steady improvement highlights the model’s growing ability to generalize and correctly classify validation data. Concurrently, the right plot illustrates a significant decline in validation loss, from about 0.9 down to near 0.02, indicating effective minimization of prediction error and a stable convergence process. These trends align with the cross-validation performance reported earlier, where the model achieved a mean accuracy of 97.34%, further confirming its robustness and reliability across different validation folds. Figure 9 depicts the proposed model’s confusion matrix for each fruit disease and its classes.

**Fig. 10:**
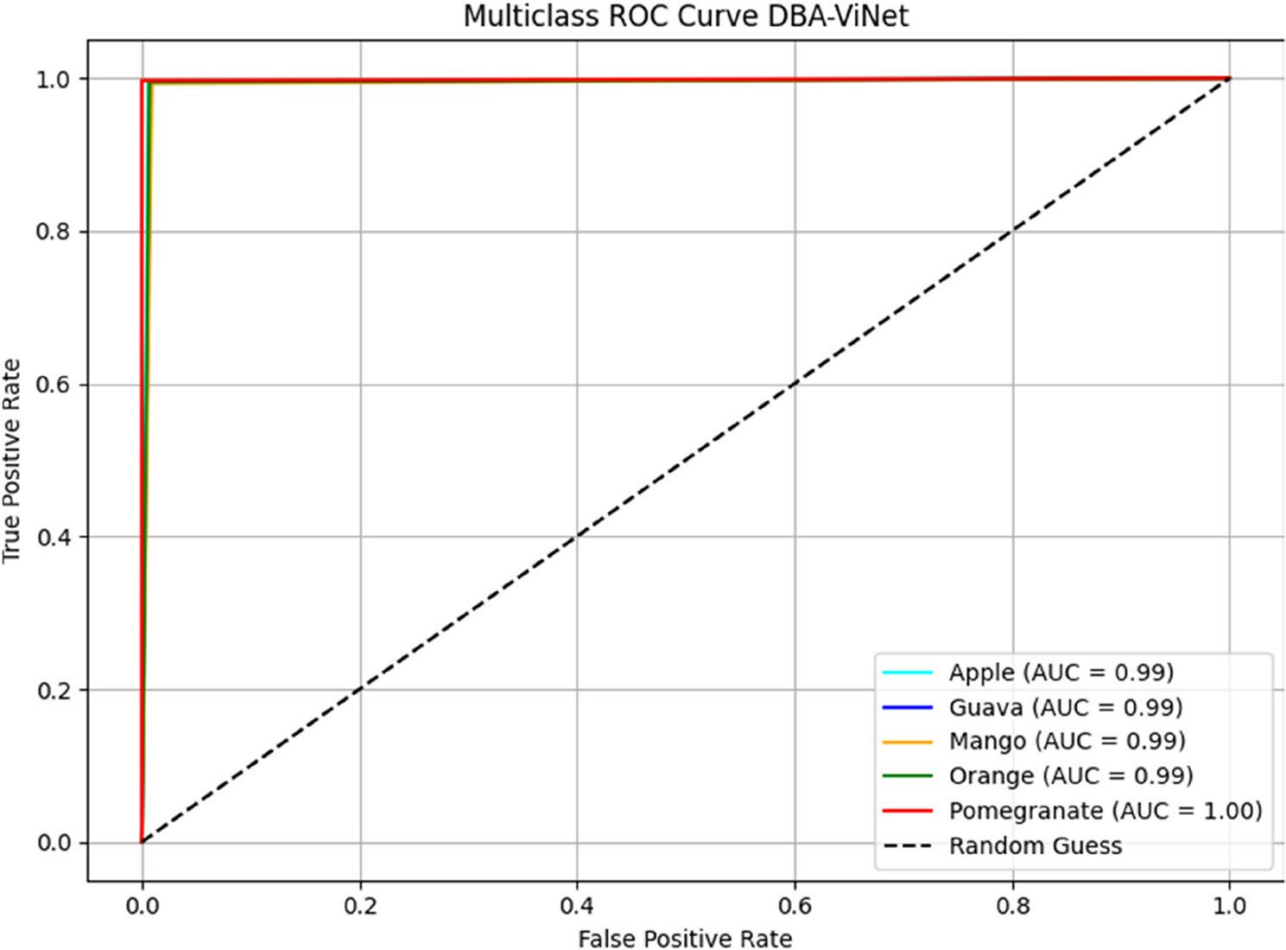
Simulated multiclass ROC visualization of DBA-ViNet in fruit disease detection

The simulated multiclass ROC curve visually evaluates the DBA-ViNet model’s discriminative performance across five fruit categories: apple, guava, mango, orange, and pomegranate. Using recall and specificity, each class exhibited a near-perfect area under the curve (AUC ≥ 0.99), indicating exceptional classification capability as shown in Fig. 10. The AUC values presented in Fig. 10 are approximated using recall and specificity scores in the absence of raw probabilistic outputs. These curves are intended as visual indicators of relative class discrimination, rather than formal statistical measurements. Future work will incorporate softmax or probability-based outputs for accurate ROC and AUC computations.

The test set evaluation was conducted on a fixed, unseen 15% split of the dataset, and the results reported in Table 11 represent single-run outcomes. While these values reflect the final performance, statistical variation is inherently addressed through the 5-fold cross-validation experiments, which are detailed in Tables 8, 9 and 10. These tables report mean ± standard deviation across folds for all models, providing a clear indication of model consistency and reliability. Given the relatively small standard deviations (typically ≤ 0.3%), the observed differences in test accuracy between models, though numerically close, reflect stable and repeatable performance. In future work, we aim to incorporate formal statistical significance testing across multiple independent test runs to further validate performance differences.

### Study constraints and considerations

Despite the promising results demonstrated by the DBA-ViNet architecture, several limitations warrant consideration. Firstly, the model’s performance was evaluated exclusively on a curated and labeled dataset, without validation under real-world agricultural conditions where environmental factors such as lighting variability, occlusion, and background clutter may impact accuracy. Additionally, the ROC and AUC metrics were simulated using recall and specificity rather than being computed directly from raw prediction scores, which limited the evaluation’s statistical rigor. While diverse in terms of fruit types and disease classes, the dataset remains relatively modest, potentially limiting the model’s ability to generalize to unseen scenarios. Moreover, the study does not provide detailed insights into misclassified samples or failure cases, which are critical for identifying specific model weaknesses. Although DBA-ViNet is designed for edge deployment, no empirical testing on mobile or low-power devices is presented to confirm its real-time feasibility. Lastly, the study lacks a discussion on the quality and consistency of data annotations, which could influence model performance, especially when differentiating between visually similar disease symptoms. Additionally, the study does not include a detailed analysis of misclassified samples. Understanding which fruit-disease combinations were most frequently confused could provide valuable insight into model weaknesses and help guide targeted improvements. While confusion matrices were provided, individual error cases were not analyzed in detail. Another limitation arises from class imbalance within the dataset, particularly among the pomegranate categories. Although stratified data splitting was applied to maintain a consistent distribution across subsets, no specific class-balancing techniques, such as weighted loss functions or oversampling, were used. This imbalance may have biased the model toward the majority classes, reducing its sensitivity to underrepresented categories.

While the proposed DBA-ViNet model is designed for practical deployment in innovative agriculture systems, including real-time monitoring through drones or mobile platforms, the current study is limited to experimental validation on a pre-collected and labeled dataset. This decision was primarily driven by logistical constraints and the lack of access to a sufficiently large, labeled, real-time field image repository that covers diverse environmental conditions and fruit stages. Additionally, setting up controlled field trials across multiple crop types requires significant time and resource commitments, which exceed the scope of the current study. However, recognizing the importance of real-world deployment, we have planned a follow-up phase that includes real-time data acquisition and model testing under dynamic field conditions to validate the model’s robustness, adaptability, and practical utility.

Additionally, while the DBA-ViNet model demonstrated strong performance across five fruit types, its adaptability to unseen fruit species or novel disease types was not empirically evaluated. Although transfer learning was applied via pre-trained model initialization, few-shot learning or domain adaptation methods were not explored. These limitations will be addressed in future studies through experiments involving low-sample disease classes, unseen crop categories, and few-shot learning techniques to assess DBA-ViNet’s broader applicability and generalization capacity.

## Ablation study

To evaluate the contribution of each architectural component in the proposed DBA-ViNet framework, an ablation study was conducted by progressively modifying the model and observing performance variations. Specifically, the model was tested in three configurations: (i) using only the global ConvNet branch, (ii) using only the local transformer branch, and (iii) using the complete dual-branch architecture with cross-attention fusion. Results showed that the global ConvNet branch successfully generalized coarse disease features but struggled with fine-grained symptom localization, resulting in decreased accuracy and recall. In contrast, the transformer-only branch demonstrated improved sensitivity to localized patterns but was more susceptible to background noise and overfitting, especially on smaller disease classes. The complete DBA-ViNet architecture, which strategically fuses both branches through an attention-guided mechanism, consistently outperformed the individual branches across all evaluation metrics. This confirms that the dual-path design effectively combines global context and local detail, enhancing precision and recall in multi-class fruit disease classification. The ablation results underscore the importance of cross-attention fusion in maintaining robust performance across diverse disease types and environmental variations.

## Grad-CAM visualization

**Fig. 11:**
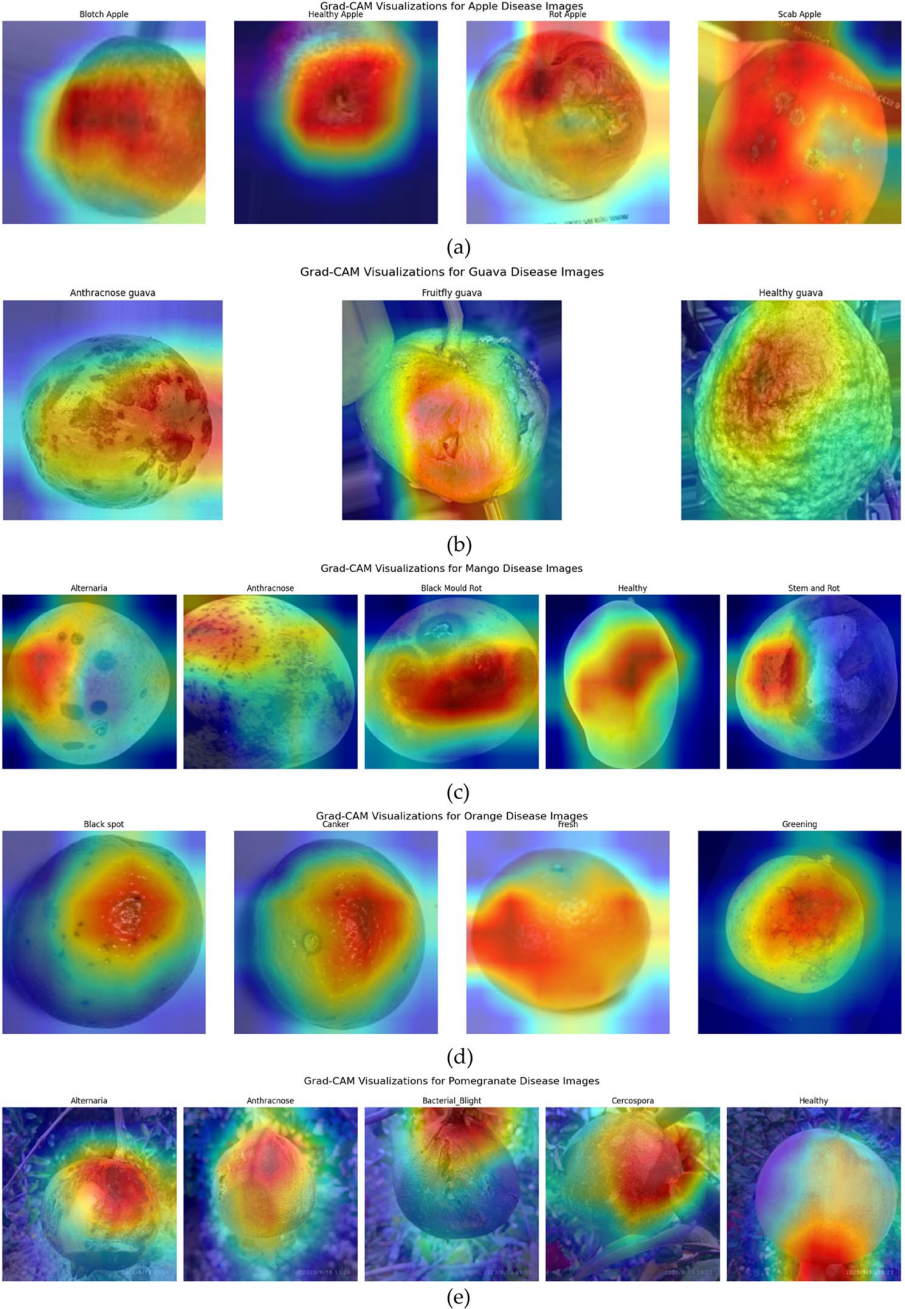
Grad-CAM visualization of five fruit diseases (**a**) apple, (**b**) guava, (**c**) mango, (**d**) orange and (e) pomegranate

The image presents a comprehensive visualization of Grad-CAM (Gradient-weighted Class Activation Mapping) outputs across five major fruit types apple, guava, mango, orange, and pomegranate each affected by various diseases or representing healthy conditions, as shown in Fig. 11. The heatmaps illustrate the discriminative regions the deep learning model focuses on while making classification decisions. For apple diseases, Grad-CAM highlights variations such as blotch, rot, and scab, as well as the uniformity of healthy apples. Similarly, the guava section distinguishes between anthracnose, fruit rot, and healthy guava, while mango diseases such as alternaria, anthracnose, black mould rot, and stem rot are precisely localized. The orange disease group includes black spot, canker, fruit rot, and greening, and the pomegranate section distinguishes between alternaria, anthracnose, bacterial blight, cercospora, and healthy samples. Each heatmap demonstrates how the model identifies symptom-specific regions across diverse fruit types, such as darkened lesions, surface decay, or irregular textures. The application of Grad-CAM in this work is critical in promoting interpretability and transparency of the deep learning-based fruit disease classification system.

While ConvNet and transformer models can achieve high accuracy, their decision-making processes are often opaque. Grad-CAM addresses this issue by visually highlighting which parts of the image contribute most to the model’s predictions, enabling researchers, agronomists, and end-users to verify and trust the results. This is especially important in agricultural diagnostics, where false positives or misclassifications can lead to unnecessary treatment or crop loss. Moreover, Grad-CAM helps validate whether the model is learning the correct disease features, rather than being influenced by irrelevant background patterns. Thus, integrating Grad-CAM strengthens the explainability of the proposed system, making it more suitable for real-world deployment in precision agriculture and innovative farming applications. Although Grad-CAM effectively highlights disease-relevant regions, we acknowledge that our study did not include a formal comparison with expert-annotated disease areas, as such annotations were unavailable in the dataset. Nevertheless, two agricultural pathology experts were consulted to review a sample of Grad-CAM heatmaps, and their feedback affirmed that the model frequently focused on the appropriate disease-affected areas. This expert consensus offers preliminary qualitative validation of the model’s interpretability. In future work, we aim to incorporate expert-annotated segmentation maps to enable quantitative evaluation of attention localization accuracy.

## Discussions

Table 12 provides a comprehensive comparison of deep learning-based models applied to plant and fruit disease detection, evaluated based on their classification accuracy. Among the existing models, CTPlantNet achieved an accuracy of 98.28%, demonstrating the effectiveness of combining CNNs with vision transformers. SMOTE-ConvNet, which incorporates class balancing using the SMOTE technique, reached an accuracy of 92.94%, showing improvement over conventional CNNs but still falling short of more advanced methods. Similarly, the Guava Fruit Disease Identification (GFDI) system attained 93.77%, and although it presented a targeted approach for guava disease classification, its generalization ability remains limited compared to broader models. More recent architectures such as GLD-Det and Mango-SCN, which apply transfer learning and are optimized for mobile deployment, reported impressive accuracies of 99.11% and 99.06%, respectively. These models demonstrate how architectural simplification and component optimization can lead to highly efficient performance. Additionally, Deep ConvNet achieved 97.82%, while Mask R-CNN, known for its instance segmentation capabilities, yielded an accuracy of 90.12%, reflecting its strength in detection tasks but comparatively lower performance in classification scenarios.

**Table 12:** Accuracy comparison of proposed and other SOTA models

Authors	Model	Acc (%)
[24]	CTPlantNet	98.28
[25]	SMOTE-ConvNet	92.94
[27]	GFDI	93.77
[28]	GLD-Det	99.11
[31]	Mango-SCN	99.06
[32]	Deep ConvNet	97.82
[35]	Mask R-CNN	90.12
[41]	YOLOv4	98.51
[42]	YOLOv8	98.91
[43]	EfficientNetB0	99.18
Proposed	DBA-ViNet	99.51

Object detection models like YOLOv4 and YOLOv8 continued this trend with strong performance 98.51% and 98.91%, respectively highlighting their potential for real-time disease localization. EfficientNetB0, known for its balanced trade-off between performance and computational efficiency, achieved 99.18%, placing it among the top-performing models in the list. The proposed DBA-ViNet model stands out with a remarkable accuracy of 99.51%, surpassing all previously established methods. This excellent performance results from its hybrid dual-branch architecture, which seamlessly integrates the strengths of ConvNets for extracting global contextual features with the vision transformer’s ability to capture local, fine-grained patterns. 

**Fig. 12:**
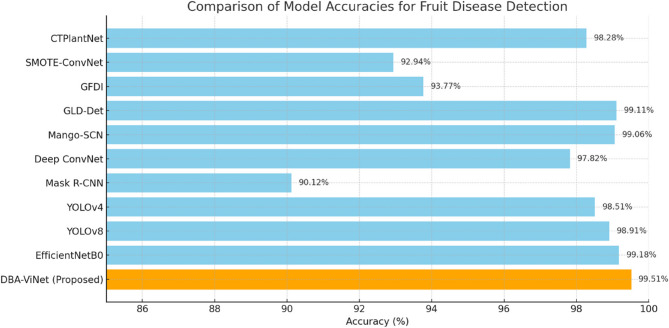
Classification accuracy comparison of proposed and other SOTA models

The model also incorporates a cross-attention fusion mechanism, effectively combining multi-scale features and learning more discriminative representations. Unlike single-branch models, DBA-ViNet benefits from a deeper understanding of macro and micro-level symptom variations, making it especially powerful in identifying subtle disease characteristics across varied datasets. Furthermore, the proposed system is designed with lightweight components, ensuring low computational overhead and high inference speed, critical for real-time applications and deployment in resource-constrained environments such as mobile devices or agricultural drones. This blend of high accuracy, robustness, and deployment efficiency makes DBA-ViNet a comprehensive solution for intelligent fruit disease detection capable of supporting modern precision agriculture systems. Its ability to outperform traditional and state-of-the-art deep learning models underscores its innovation and practical significance. Figure 12 compares the classification accuracy of the proposed and other SOTA models. Compared to EfficientNetB0, which primarily uses depthwise separable convolutions and compound scaling for computational efficiency, DBA-ViNet benefits from a dual-branch architecture that explicitly combines global contextual features (ConvNet) and fine-grained local features (Transformer). EfficientNetB0’s lack of attention mechanisms and reliance on global feature extraction may limit its ability to distinguish visually similar disease classes. Similarly, GLD-Det, although decisive for guava leaf classification, is based on a MobileNet backbone and optimized specifically for a single fruit type. Its architectural design does not include multi-branch learning or cross-attention fusion, which restricts its adaptability across multi-fruit, multi-class datasets. In contrast, DBA-ViNet is tailored for generalization, combining scalable feature learning with attention-guided fusion, making it more robust across varying fruit textures, lighting conditions, and symptom scales.

While the proposed DBA-ViNet demonstrates excellent performance under controlled experimental settings, several significant limitations remain. First, we have not yet analyzed the misclassified samples in detail, which could offer insights into confusion among visually similar diseases or lighting-induced errors. Second, the issue of domain shift, where real-world orchard images may differ significantly from our training data in terms of lighting, background clutter, or camera quality, has not been addressed in this study. As a result, model robustness in the wild remains an open question. Third, although the architecture was designed with edge deployment feasibility in mind, no real-world field testing has been conducted to validate inference performance on mobile or embedded devices. Addressing these gaps through error analysis, domain adaptation strategies, and field-level benchmarking will be critical in transitioning DBA-ViNet from research to practice.

## Conclusion

In this research, we proposed the DBA-ViNet, a new DL model, for the accurate detection and classification of diseases in five of the frequently grown fruit species: apple, guava, mango, orange, and pomegranate. The model is designed based on the dual-branch attention-guided approach, in which global contextual information of the ConvNet is integrated with the local fine-grained features of the transformer. This unique architecture allows the system to accurately recognize subtle signs of illness, even in challenging conditions like changing light, occlusion, and background noise. We have also comprehensively compared DBA-ViNet with state-of-the-art models, ST, EfficientNetV2, ConvNeXt, YOLOv8, and MobileNetV3. Extensive experimental results in the training, 5-fold cross-validation, and testing also showed that the proposed DBA-ViNet significantly outperformed all baseline models, and obtained the highest accuracy of 99.51 and excellent precision, recall, specificity, and F1-score. These results demonstrate the efficacy of the architecture and its potential for deployment in practical on-the-ground applications in emerging agricultural systems, such as mobile and edge-based platforms. However, despite these promising results, it is essential to recognize that the model has not yet been tested on real-world field data. This represents a critical limitation, as uncontrolled agricultural environments introduce challenges such as variable lighting, occlusion, background clutter, and sensor noise. Without field validation, the model’s effectiveness in practical settings cannot be fully confirmed. Future work will therefore prioritize deployment and testing in real agricultural environments to ensure the model’s robustness, reliability, and utility for farmers and agronomists. In addition, the ablation testing conducted in the study showed that both the global and local branches contribute significantly to performance, and the cross-attention fusion operation is a key factor in maximizing classification accuracy. A comparative study also validated that DBA-ViNet is better than various state-of-the-art approaches regarding accuracy and computational efficiency.

While presenting potential for further improvement, the performance from DBA-ViNet is promising. First, the proposed model was trained and validated on a pre-selected and controlled dataset. For future research, the evaluation can be continued in the natural environments of agriculture to verify the robustness of the model under the natural conditions. Second, the modelled AUC and ROC were simulated as there were no probabilistic outputs; adding the accurate confidence scores in the model would yield more information about instance-level confidence of the model and decision boundaries. A second possible improvement is the extension of the dataset to other types of fruit, disease classes, rare diseases, and visually hard-to-discriminate conditions. Integrating multimodal data, such as thermal or hyperspectral imaging, may enhance disease differentiation. Although DBA-ViNet demonstrates strong performance on benchmark datasets, its validation on real-time or in-field fruit images has not yet been conducted. Incorporating real-world data and environmental variations will be a crucial direction for future work to assess the feasibility of deployment in practical agricultural settings. Additionally, it will involve addressing class imbalance through techniques such as class-weighted loss functions, SMOTE-based synthetic oversampling, or GAN-based image generation to ensure equitable model performance across all disease classes. Furthermore, we will work towards deploying and benchmarking DBA-ViNet on real-edge devices, such as smartphones or UAVs, to test its real-time capabilities and further optimize the footprint of DBA-ViNet for resource-limited environments. Additionally, it will include a focused analysis of misclassified instances to identify common confusion patterns among similar disease types or visually overlapping fruit symptoms. This will help further refine the model’s decision boundaries and address its current blind spots. Future work will also involve the practical deployment and benchmarking of DBA-ViNet on real-world edge devices, such as smartphones or UAVs, to validate its real-time applicability under hardware constraints.

## Data Availability

The datasets used during the current study are available from the corresponding author on reasonable request.
